# Unraveling of a Strongly Correlated Dynamical Network of Residues Controlling the Permeation of Potassium in KcsA Ion Channel

**DOI:** 10.3390/e23010072

**Published:** 2021-01-06

**Authors:** Salvatore M. Cosseddu, Eunju Julia Choe, Igor A. Khovanov

**Affiliations:** School of Engineering, University of Warwick, Coventry CV4 7AL, UK; salvatore.cosseddu@gmail.com (S.M.C.); e.j.choe.00@gmail.com (E.J.C.)

**Keywords:** ion channels, protein dynamics, molecular dynamics

## Abstract

The complicated patterns of the single-channel currents in potassium ion channel KcsA are governed by the structural variability of the selectivity filter. A comparative analysis of the dynamics of the wild type KcsA channel and several of its mutants showing different conducting patterns was performed. A strongly correlated dynamical network of interacting residues is found to play a key role in regulating the state of the wild type channel. The network is centered on the aspartate D80 which plays the role of a hub by strong interacting via hydrogen bonds with residues E71, R64, R89, and W67. Residue D80 also affects the selectivity filter via its backbones. This network further compromises ions and water molecules located inside the channel that results in the mutual influence: the permeation depends on the configuration of residues in the network, and the dynamics of network’s residues depends on locations of ions and water molecules inside the selectivity filter. Some features of the network provide a further understanding of experimental results describing the KcsA activity. In particular, the necessity of anionic lipids to be present for functioning the channel is explained by the interaction between the lipids and the arginine residues R64 and R89 that prevents destabilizing the structure of the selectivity filter.

## 1. Introduction

Over the last few decades, the bacterial K${}^{+}$ ion channel KcsA [1] found in *Streptomyces lividans* has been widely studied in order to understand the structural and functional features of potassium ion channels. It continues to be of interest [2,3,4,5,6,7,8] in part due to its sequence similarity to eukaryotic K${}^{+}$ channels, and in part because of its role as an archetype for ion permeation, selectivity, and the complex interplay of the different “gates” which governs a variety of current patterns observed experimentally in the K${}^{+}$ channel superfamily [9,10,11]. These patterns are defined by small structural rearrangements of the pore region once the inner gate is opened [4,10,11,12,13]. The local rearrangements are mostly obscure as current experimental techniques are unable to provide the combination of spatial and temporal resolution needed to identify the underlying atomistic-level mechanisms. Structural studies showed that the current patterns depend on a number of residues, some of which are located relatively far from the pathway of K${}^{+}$ permeation, and physiological recordings revealed the strong influence of the K${}^{+}$ concentration in the outer bulk on the patterns [1,10,14,15,16,17,18,19]. The anionic phospholipids modulate the function of the channel [20,21,22] and the addition of phosphatidic acid lipid significantly affects the permeation [23].

As with most of the K${}^{+}$ ion channels, KcsA contains a highly conserved amino acid sequence motif TXXTXGYGD known as the *signature sequence*, which corresponds to residues 75 to 79 in the reference X-ray structure 1K4C [1], where “X” in position 76 is replaced by valine. The whole quaternary structure of KcsA is divided into three functional regions: the selectivity filter, a water-filled cavity, and an inner gate associated with large movements in the transmembrane helices for opening the channel [16,24,25,26,27]. The selectivity filter (SF) is the narrowest part of the pore. The SF consists of five well-defined binding sites for K${}^{+}$ ions by exposing the backbone carbonyl groups of the residues toward the channel axis [28]. These sites are commonly labeled as S0 (below T75), S1 (between T75 and V76), S2 (between V76 and G77), S3 (between G77 and Y78), and S4 (between Y78 and G79). The permeation is forced to occur in a single file fashion as a hopping of an ion from one site to another site.

The filter plays a role in both ion selectivity and modulating the current. The latter corresponds to random-like switching (gating) between zero and finite values of the current. Once the inner gate is opened, the current is regulated by small structural rearrangements. They are responsible for different gating processes, such as the *C-type inactivation* and the *modal-gating*, from which complex patterns of ion current arise [13,14,16,17,28,29,30]. The C-type inactivation corresponds to very long inactive (zero current) time intervals under steady-state conditions. The modal gating is associated with three different modes of the single-channel currents in KcsA. Two modes correspond to a high and low probability of the pore to be in the conducting (active) state, respectively. Third mode is a high-frequency flicker mode representing in bursts of fast switching back and forth between active and inactive states [13,14].

The inactivation in the KcsA channel is a common feature in functioning potassium channels, including eukaryotic ones [10]. Therefore, the C-type inactivation has been extensively studied using a variety of different experimental techniques such as crystallography, NMR, ssNMR, fluorescence measurements, and computational studies, leading to several hypotheses reflected in the recent detailed review [6]. A combination of structural (X-ray) studies and physiological measurements of the wild type (WT) of KcsA and its different mutants suggests that several residues behind the SF could be involved in filter’s structural rearrangements during the inactivation [9,14,15,18,19,29,30,31,32,33]. These studies led to the suggestion of four channel’s states with an open or closed inner gate and a conducting or non-conducting SF [4,11]. One of the hypotheses [6,11,34] suggests that the activation by opening the inner gate simultaneously alters the SF via allosteric coupling [35,36]. This coupling leads to a slow (on a time scale of seconds) collapse of the SF to a non-conducting configuration. Although structures corresponding to an inactive channel with closed and open inner gate were reported [11,28,30], a structure of an active channel with an open gate and a conducting SF is still missing. Note that the canonical structure 1K4C [28] with a conductive configuration of the SF has a closed inner gate. Another set of experiments used mutagenesis of residues in the SF and demonstrated that the ion occupancy in specific sites controls the inactivation [37,38,39]. This result leads to the second hypothesis that the SF alone could play the role of an “inactivating gate” without the involvement of the inner gate [6]. This hypothesis tightly links to experimental observations that the SF’s conformational dynamics in the WT KcsA and its mutants govern gating properties in the KcsA channel [14,40,41]. Although these two hypotheses are sometimes considered controversial [6,42], they could coexist and reflect the complexity of the KcsA channel.

An additional complication to this gating-permeation picture is the dependence of the K${}^{+}$ current and the filter rearrangements on the extracellular K${}^{+}$ concentrations, common among numerous K${}^{+}$ channels [9,10,38,43,44]. The probability of the inactivation grows with decreasing K${}^{+}$ concentration. This effect has been suggested to link to a “foot-in-the-door” mechanism in which an ion resident in the filter stabilizes the conductive conformation and reduces the inactivation probability [9,16,38]. The exact location of the binding site responsible for the effect is unknown. However, it is suggested such site can be located either at the extracellular mouth or in the central region of the selectivity filter [10,38,45].

In the majority of these studies static (crystallographic) X-ray structures were used for describing the function. However, these static pictures do not provide details of the essentially dynamical picture of the inactivation. Therefore, general mechanistic knowledge of the gating behavior, which comprises transitions between various states, remains obscure [4,10,11,13]. Recent applications of solid-state NMR [41], 2D IT spectroscopy [2], and florescences measurements [46] for analyzing channel dynamics could address the uncertainties in functional relevance of crystallographic structures. However, a mechanistic picture of the filter’s rearrangements with simultaneous dynamical analysis of ions and water molecules is beyond the current experimental techniques. Molecular dynamics (MD) simulations offer valuable tools for exploring dynamical properties at the atomic level [47,48,49]. For example, MD helped discriminate between “knock-on” and “snug-fit” mechanisms of the permeation in the KcsA channel [50]. In turn, the structural study [51] recently resolved some controversy in MD simulations [2,3] on water involvement in knock-on mechanisms.

The inactivation hypotheses were also discussed by applying MD approaches [34,52]. These computational studies concluded that the activation via opening the inner gate affects the low site S0 in the SF by enhancing the permeation [34] and controlling the SF’s stability [52]. The latter result leads to new perspectives [53] for the inactivation mechanism as a process tightly controlled by the inner gate, which could be in different partially open states [52]. The dynamics near SF becomes less important in this picture. A quick collapse of the SF in the case of a widely open inner gate was observed [52]. The collapse happens on much shorter than broader time scales of the inactivation, which can be the order of seconds. The same time scale for collapsing the SF was reported in recent unbiased simulations of a similar open structure [54]. The former result [34] partly supports these new perspectives as it shows that configurations of the inner gate affect the permeation. However, a collapsed SF has not been reported for the performed biased MD simulations for the open inner gate [34]. As mentioned above, a crystallographic structure of the KcsA channel with a conducting SF and an open inner gate is not available, so such a structure was created in silico [34,52] using combinations of the reported structures [55]. Differences in structures used for creating proteins with an open inner gate could explain some contradictions in those MD approaches [34,52].

Heer et al. [34] also reported that the permeation barrier in the canonical (a conducting SF and a closed inner gate) structure 1K4C [1] is too high to consider its SF configuration as conducting. This conclusion was derived from biased simulations using the umbrella sampling method [56]. The obtained barrier was found to be too high for observing the permeation rate according to experimental recordings [57]. This result is in line with the work reported earlier by Fowler et al. [58]. In contrast, other unbiased MD simulations [59,60,61] confirmed the conducting state of SF. Note that the SF of structure 1K4C was used in the majority of the simulations mentioned above. Two major factors could explain such discrepancies. The first factor is the use of either biased or unbiased MD approaches. The second factor is defined by differences between obtained in silico structures with an open inner gate. While generating a new structure in silico applied a tight control of SF backbones and ions’ and water molecules’ locations, other residues were not over-sighted. In biased approaches, just one or two so-called collective variables (typically ions locations) were considered assuming that the dynamics of all other variables (water molecules and residues) can be averaged out. Yet, in unbiased approaches some constraints are applied on the protein during MD simulations.

Thus, conformations and behavior of many residues, especially in the region of the SF, were kept out of the consideration despite the experimental studies that identified a number of residues strongly altering the inactivation and gating [9,14,15,18,19,29,30,31,32,33]. A series of papers by Cordero et al. [9,14,29] suggested that the stability of the SF depends on a hydrogen-bond (H-bond) network formed by the triad of residues E71-D80-W67. In particular, the substitution of glutamate E71 with alanine A71 suppresses the inactivation, and the conduction is observed even in low K${}^{+}$ concentrations [14]. Therefore, there is a gap in understanding how states of this triad are linked to the permeation. In this manuscript, we aim to provide a mechanistic picture of rearrangements in the WT KcsA protein and discuss the mechanisms by which residues behind the SF interacts with the backbones of the SF, and ions and water molecules within. This picture is an essential piece of the inactivation puzzle and in addressing issues of MD biased simulations.

**Figure 1:**
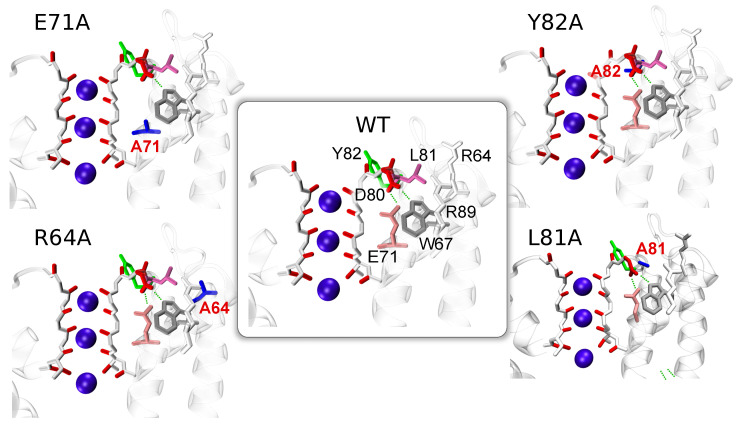
A region near the SF in the different proteins: WT, E71A, Y82A, R64A, and L81A, is shown. With the exception of the mutated residues, the other residues are in the X-ray conformation [28]. Ions are shown as purple spheres interacting with oxygen atoms (red color) of residues in the SF. The key residues are highlighted by different colors, mutated residues are shown in blue.

A large number of residues in the SF region of the KcsA protein means that a brute force (combinatoric) consideration of all possible combinations of different residues states is unrealistic. The state-of-the-art microseconds MD simulations [52,54] show that structure 1KC4 adapts one of the multistable states and no rearrangements of residues behind the SF were reported. In the present work, therefore, we first conduct a comparative analysis between the WT protein and different mutants (E71A, Y82A, R64A, and L81A) (see Figure 1) where key residues are replaced by the short, weakly interacting alanine. The selection of the mutated structures is based on previous experiments [9,14,15,18,19,29,30,31,32,33] which reported different probability of the inactivation. MD simulations were combined with biased free-energy methods, well-tempered metadynamics [62], and statistical analysis. The biased simulations introduce additional perturbations into the protein and, therefore, verify the stability and thermodynamics of different states of the SF. The results of MD simulations are critically assessed against published experimental and computational investigations. The study was designed to unveil the complex dynamics that underlie the permeation path in the WT KcsA protein and has allowed us to identify a cooperative network of dynamically interacting residues located near the SF. Note that preliminary results of this study were reported in work [63].

In this paper, first, an analysis of residues’ dynamics in mutated structure E71A is presented. The relationship between conformational changes at the SF and rearrangements of residue D80, located at the channel’s outer entrance, is explored. Second, a network of residues, which affect the ion permeation, is identified by comparing the dynamics of proteins WT, Y82A, R64A and L81A. Third, a thorough description of the network dynamics, including energetics of transitions in the network, and its influence on the filter structure and the ion permeation is presented.

## 2. Methods

### 2.1. Setup of the Simulations

The simulations were performed using NAMD 2.8 and 2.9 [64] in the NPT ensemble with pressure 1.01 bar and temperature 310 K. A multiple timestep algorithm was used [65,66]. In the case of unbiased simulations the integration step size was 1 fs, nonbonded nonelectrostatic interactions were calculated every 2 fs, and electrostatic forces [67] every 4 fs. In biased simulations, the step size was 2 fs, nonbonded nonelectrostatic interactions were calculated every 2 fs, and electrostatic forces every 6 fs. The CHARMM27 force field (FF) was used for the protein, with a modification in the Lennard–Jones term to represent the interaction between K${}^{+}$ and the carbonyl oxygens of the protein, CHARMM36 for the lipids, and TIP3P for water were applied [50,68,69,70,71,72]. The system was prepared by embedding the X-ray structure (pdb code 1K4C; solved at 2 Å resolution [28]) with 2 K${}^{+}$ in the SF and 1 K${}^{+}$ in the cavity, in a membrane patch of 222 molecules of 1-palmitoyl-2-oleoylphosphatidylcholine (POPC), and solvated by 17740 water molecules [73,74,75]. A potassium concentration in the aqueous phase of 0.2 M was obtained with 63 K${}^{+}$ ions, and the system was neutralized by 75 Cl${}^{-}$ ions. The ions were distributed over the whole simulation box. Relaxation of the system and preparation of the mutants is described in Supplementary Materials.

Coordinates, if not otherwise stated, were considered every 2 ps, ignoring an initial equilibration period of 1 ns.

### 2.2. Collective Variables and Order Parameters

Collective variables (order parameters) used in this work are defined as follows. (i) Variables $\psi_{76}$ and $\psi_{81}$ are the ψ dihedral angles measured for residues indicated in the subscripts, and they follow the standard definition. (ii) Variable $\chi 1_{81}$ is the χ1 dihedral angle of the L81 residue, which follows the standard definition as well. (iii) Variable SC${}_{80}$ is the position of the D80 side chain considered as the distance between C${}_{\gamma }$ atom of D80 and a reference atom, C${}_{\alpha }$ of A73. Note that the latter residue shows the lowest fluctuations in RMSD analysis. (iv) The distance D80–R89 is between C${}_{\gamma }$ atom of D80 and the C${}_{\zeta }$ atom of the closest R89 residue in the quaternary structure. (v) SF length, the length of the TVGYG sequence, is measured as the distance between the C${}_{\alpha }$ atoms of residues T75 and G79. (vi) The distance R64–SF is measured between C${}_{\zeta }$ atom of R64 and the center of mass (COM) of the selectivity filter. (vii) The distance E71–D80 is between C${}_{\gamma }$ atom of D80 and the H-bond donor oxygen of E71. (viii) The coordinates $z_{K1}$ and $z_{K2}$ are the *z* coordinates of the K${}^{+}$ ions bound to the filter (ions labeled as K1 and K2 in Figure 5); the coordinate system has been centered with respect of the COM of the SF, in order to remove the components associated with the protein diffusion in the membrane.

The COM of the SF was defined by the atoms N, C${}_{\alpha }$, and C of residues from 74 to 78 of all four subunits.

### 2.3. Free Energy Calculations—Metadynamics

Different approaches are used to enhance the sampling when high energetic barriers between states do not allow an appropriate sampling for the investigation of rare events and the reconstruction of the free energies. These are often based on non-Boltzmann sampling.

Well-tempered metadynamics (wt-metaD) is a non-Boltzmann sampling method based on a history-dependent bias potential, created as a sum of Gaussians centered along the trajectory of specified collective variables (CVs) [62,76,77]. In wt-metaD technique, the height of Gaussians added is history-dependent, and this dependence is associated with a parameter ΔT having the dimension of temperature. This parameter was adjusted for each simulation. The NAMD package [64] includes module *colvar* for performing wt-metaD. Additional details of the implementation of wt-metaD and the selection of the relevant parameters are reported in the Supplementary Materials, section “Well-tempered Metadynamics”.

### 2.4. Initialization of WT-R64D80 Simulation

For the simulation denoted as WT-R64D80, an equilibrated conformation of WT KcsA simulated for 6 ns was used. During first 20 ps of the relaxation, residues L81 and R64 were restrained. Every residue L81 was restrained towards the flipped state by the harmonic potential with a spring constant of 24 kcal/mol degree${}^{2}$ and centered on $185^{\circ }$. The harmonic potential (spring constant 20 kcal/mol degree${}^{2}$ centered on $-160^{\circ }$) was applied on χ1 dihedral angle of each R64. Note that the latter restraints were added to speed up the calculation, but are not strictly necessary to obtain the desired configuration. A further 25 ps of relaxation were performed without any restraint.

### 2.5. Statistical Analysis

The statistical analyses were performed using VMD 1.9 [78] and R software environment [79]. Several packages for R were used in addition to the core functions: bio3d, ggplot2, car, and MASS [80,81,82,83,84].

All the free-energy surfaces (FES) presented in this work were smoothed via cubic smoothing spline (grid length 80) and thin plate spline methods (grid sizes 80×80) which are implemented in R packages stats v2.15.3 [79] and fields v6.7.6 [85], respectively.

## 3. Comparative Analysis of Dynamics of WT and Mutated Proteins

### 3.1. Considered Proteins

The simulations commenced from relaxed systems, prepared from the X-ray structure solved at 2 Å resolution [28], as explained in the previous section “Methods”. The KcsA channel has a tetrameric structure, and the four subunits of the KcsA are referred by capital letters A, B, C, and D. The SF is described as a five-site pore [24,25] through which ions and water molecules move in a single-file fashion. The standard notation of the sites is used: S0 to S4 starting from the outer site. The configurations of the SF are described by a five-character string (from S0 to S4), where a “K” represents a K${}^{+}$ ion, “w” a water molecule, “0” a vacancy; when a K${}^{+}$ is present in the cavity a “K” is appended, separated from the filter occupancy by sign “+”. For example, the configuration wKwKw+K means the presence of K${}^{+}$ ions in S1, S3, and the cavity separated by water molecules. In comparison, KwK0K implies the presence of K${}^{+}$ ions in S0, S2, and S4, a water molecule in S1, a vacancy in S3 and no a K${}^{+}$ ion in the cavity. Consistent to the previous literature [14,86], the results are described by considering the extracellular region as an outer region and “up” in the frame of reference, while the intracellular region is considered as inner and “down”.

Among the numerous mutants, which differ from the WT in the gating behavior, three proteins have been considered: (i) E71A is be resistant to the inactivation, (ii) R64A shows a sharp reduction of the inactivation, and (iii) Y82A demonstrates an enhancement of the rate and extent of the inactivation [14]. It is, therefore, possible to specify a trend in the inactivation probability of these proteins: E71A < R64A < WT < Y82A. An additional mutant, L81A, was created for testing the roles of residues L81 and R64, and their coupled motions.

### 3.2. Dynamics of Mutant E71A

The link between residues E71 and D80 is considered to be an important one for KcsA functioning. A special patch in the force fields was introduced to tune the link for observing ions’ conduction [86]. However, the mutation of glutamate (E71) to alanine (A71) does not affect the conductivity and, moreover, it suppresses the inactivation. This observation means that other residues play an essential role to keep the SF in a conducting configuration.

The mutation by replacing glutamate E in position 71 by alanine A results in the structure E71A which was studied experimentally by Cordero et al. [14]. The authors demonstrated that the permeation path undergoes large conformational rearrangements in the non-inactivating mutant E71A. The rearrangements primarily occur in the region of V76 residue. Additionally, the authors [14] reported a strong upward movement of residue D80 relative to its position in the WT structure, leading to the “flipped E71A” structure.

For understanding the influence of the mutation on the dynamics and interactions of residues, an unbiased MD simulation of length 24 ns was performed. Several rearrangements in the permeation path were observed during the simulation. The most noticeable changes happened among the residues of the TVGYG sequence in the subunit B. Rotation of the V76-G77 peptide group occurred at 9 ns, and the rotation remained stable until the end of the simulation. Similar transitions have been reported in the literature for both WT and E71A. Many hypotheses [30,58,87,88,89,90] have been proposed for explaining the transitions which are usually referred to as “flipping of V76”. However, understanding the significance and origin of the transitions is still missing.

The flipping of V76 has been suggested by different authors to be able to generate non-conductive conformations associated with the C-type inactivation or flicker mode [30,88]. We performed various permeation tests on V76 flipped conformations of E71A and WT by performing unbiased simulations with two ions in the cavity (see Supplementary Materials, section “Permeation in the V76 flipped configurations of E71A and WT”). The simulations revealed that reverse transitions of V76 occurring easily in the case of K${}^{+}$ permeation. This result supports the hypothesis of Domene et al. [89] that flipping of V76 is not responsible for the C-type inactivation. Furthermore, the observed conductivity suggests that the flipping of V76 alone is not sufficient even for short-living inactive states, which are associated to the modal-gating, and that additional conformational readjustments are necessary for generating meta-stable non-conductive states.

**Figure 2:**
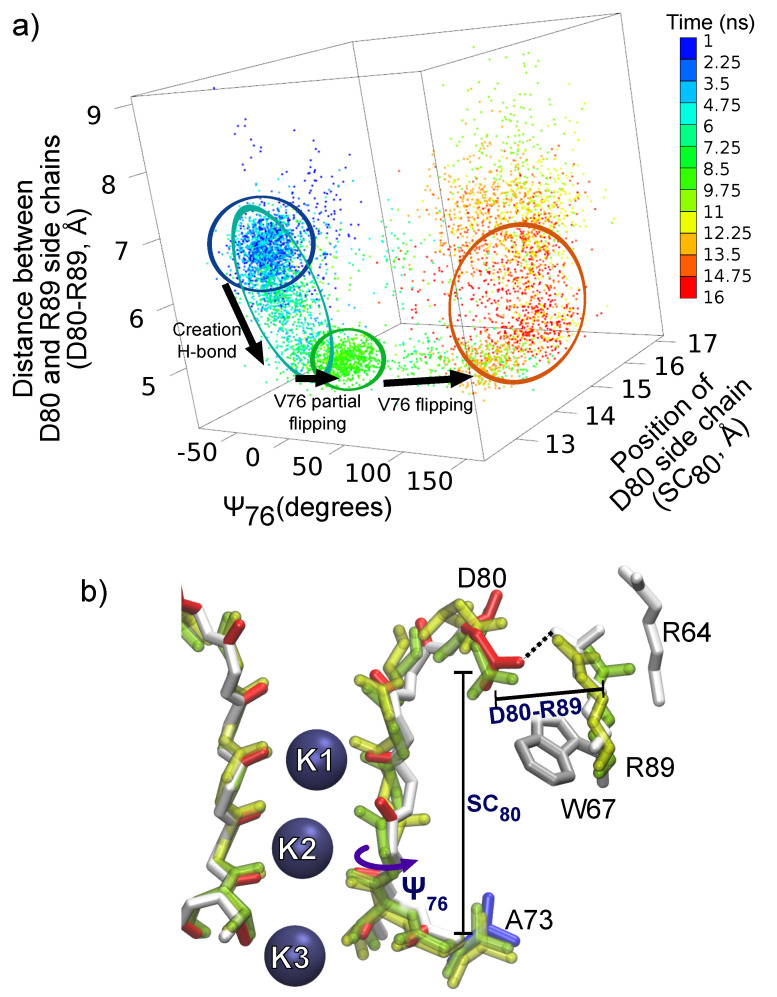
The influence of residues D80 and R89 on structural rearrangements in mutant E71A. (**a**) The evolution of the system (subunit B, initial 1 ns ignored as the relaxation interval) revealed that the stress induced by the D80–R89 H-bond led to rearrangements in the filter structure (the flipping of V76) and to an outward transition of D80. The conformational space is defined by a set of the order parameters (see “Methods”): (i) SC${}_{80}$ the position of side chain D80; (ii) D80–R89 distance, where residue R89 belongs to the neighboring subunit; and (iii) $\psi_{76}$. Time evolution of the system in the conformational space is coded by color scale shown in the colorbar. (**b**) Superposition of snapshots from the simulation of E71A: An initial configuration (green drawing); a configuration with the D80–R89 H-bond and partially flipped V76 (colored drawing); and a configuration at the end of the simulation (yellow drawing).

The simulation of E71A showed that interactions between D80 and an arginine nearby (R89) could trigger structural rearrangements of the filter. D80 side chains, which are negatively charged, demonstrated relatively large fluctuations towards the extracellular region (see Supplementary Materials, Figure S2). These fluctuations are promoted by strong inter-domain electrostatic interactions with positively charged arginines R89. The interaction between the residues D80 and R89, and the corresponding rearrangements of the SF, are illustrated in Figure 2. The conformational space in Figure 2a is defined by the three order parameters (conformational changes for subunit B only are shown): (i) the dihedral angle ψ of V76 ($\psi_{76}$); (ii) the position of the D80 side chain (SC${}_{80}$); (iii) the distance between D80 and nearest R89 (D80–R89). Initially, the dynamics of D80 and R89 appear uncorrelated (blue clouds). Some correlations arose (light blue cloud) as the time advanced because of an intermittent creation of a H-bond (D80–R89 distance is around 3 Å) between D80 and R89. Note that similar H-bonds between D80 and R89 have been reported in the literature also occurring for the WT structure [91]. The presence of the D80–R89 H-bond in E71A protein is associated with a small drift in the position of D80, SC${}_{80}$ is changed from 13.5 to 13.8 Å (see Supplementary Materials, Figures S3 and S4, for more details). The temporary strengthening and stabilization of the H-bond was accompanied by a distortion of the filter structure (in Figure 2a clouds blue to green, and in Figure 2b structure green to colored). Residue V76 assumed a *partially flipped* conformation ($\psi_{76}\approx 50^{\circ }$) in the distorted structure. This observation is an important result since it demonstrates that the backbone structure of the sequence GYGD is rigid enough for delivering a perturbation from D80 to the V76-G77 peptide group. It is shown below that the rigidity of the GYGD backbone strongly affects the SF flexibility.

Time series reported in Supplementary Materials (Figure S3) further demonstrated that, in turn, V76 partial flipping affected the permeating K${}^{+}$ ions, causing an inward shift of the outermost ion K1. Thus, ions’ dynamics are linked with the dynamics of residues behind the filter, D80 and R89. The partially flipped conformation of V76 appears to be unstable and evolved into a complete flipping of V76. The D80–R89 H-bond caused additional small transitions in the TVGYGD sequence until a slight movement of the D80 towards the extracellular side (see Figure S3 in Supplementary Materials and Figure 2b colored to yellow) restored the initial uncorrelated motions of the D80 and R89 (red clouds in Figure 2a) causing a breakage of the H-bond.

Although the described path is one among many available toward a V76 flipped configuration in protein E71A, these results demonstrate that the creation of H-bonds with residue D80 can trigger structural rearrangements which propagate to the filter because of the relative rigidity of the GYGD sequence backbone. The arginine R89 is able to promote the triggering transitions by creating a strong H-bond with residue D80. In the following sections, further evidence is presented for confirming that all the residues which can form H-bonds with D80 play a significant role in conformational rearrangements of the permeation path.

### 3.3. Correlated Dynamics of L81 and R64 Residues

The results of the previous subsection indicate that residue E71 plays an essential role in the inactivation and, therefore, in the dynamics of the WT protein. In mutant E71A, alanine in position 71 does not form bonds with D80 and residue D80 is very flexible. In contrast, in the WT protein, residue D80 is restrained by a strong link between D80 and E71. For the identification of residues that affect the permeation path, we performed a comparative analysis of three different proteins in which E71 is present. The selected proteins are the WT protein and mutants Y82A and R64A. These mutants show distinct behaviors for the inactivation: Y82A has significantly higher, and R64A has reduced the inactivation probability in comparison to WT. All three structure were simulated starting with the same initial configuration (excluding mutated residues) for different but comparable intervals: 38 ns, 28.5 ns and 23 ns for WT, Y82A and R64A respectively. Note that in the WT protein, residue R64 directly interacts with L81, which is a neighbor of residue Y82 (Figure 1).

The root mean square displacements (RMSDs) of the backbone atoms of each residue reveal residues which showed different behaviors across the three selected proteins; the X-ray structure of WT was used as the reference [28]. The results are reported in Figure S5 in the Supplementary Materials. The RMSDs analysis shows that fluctuations of the arginine R89 are wider in the proteins with a higher probability of inactivation, WT and Y82A, than in R64A. This observation additionally supports the hypothesis of a particular role of this arginine in the conformational variability of the pore. However, residue R89 in proteins WT and Y82A show similar RMSDs, and the difference in RMSDs of R89 in R64A and WT structures is relatively small. These facts imply that the dynamics of R69 by itself cannot account for the substantial diversity in the inactivation between these three proteins.

**Figure 3:**
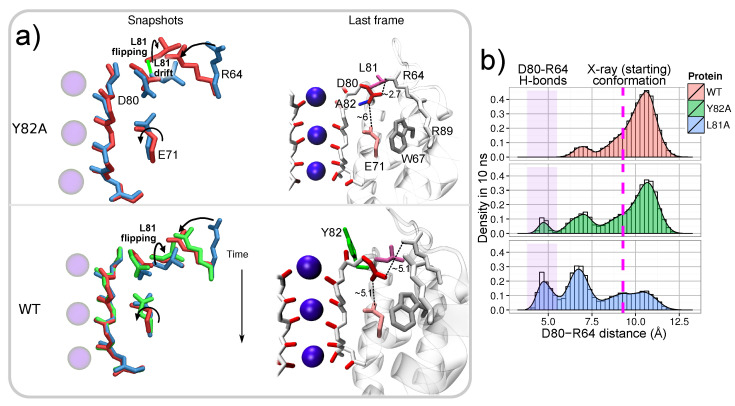
(**a**) The disruption of E71–D80–W67 linkages in Y82A and WT, promoted by the D80–R64 interaction. Snapshots and the final configurations for simulations of Y82A and WT are reported. Residues in the snapshots are superimposed with respect of the heavy atoms of the SF. The color sequence in the snapshots is (i) blue, an initial state; (ii) green, a transition state (distinguished only for the WT and characterized by the E71 χ1 angle of 120 degrees); and (iii) red, E71-D80-W71 linkages disrupted. Distances in the figures are reported in Å. (**b**) Comparison of the probability density of the D80–R64 distance in WT, Y82A, and L81A calculated for 10 ns of simulation. The initial D80–R64 distance is 9.3 Å and shown with the dashed magenta line. Distance D80–R64 which is less than 5.2 Å indicates the formation of the D80–R64 H-bond. Data from all the four subunits were used.

A closer inspection reveals the importance of second arginine residue, R64, which has relatively large RMSDs in WT and Y82A. The mutation of this arginine with alanine in structure R64A leads to a significant reduction of the RMSDs of the residue in position 64. In WT and Y82A proteins, arginine R64 can approach and interact with D80 and create strong H-bonds similarly to R89 in mutant E71A (Figure 3a). The possibility of a H-bond between R64 and D80 is important considering that R64 is located relatively far from D80 in the static structure provided by X-ray experiments [28] (D80-R64 distance =9.3 Å). Residue R64 fluctuated over wide ranges and, more importantly, it can destabilize linkages between the triad of E71-D80-W67 via the interaction with residue D80 (Figure 3a). This interaction occurs more prominently in mutant Y82A, the simulation of which ended with a broken triad E71-D80-W67 in two subunits. As a result of the R64–D80 interaction, residue D80 can rotate around the dihedral angle χ1 and such rotations were observed a few times during simulations (see Figure S6 in the Supplementary Materials). In Y82A and WT proteins, the flexibility of D80 promoted by R64 leads to several multistable configurations, one of which includes a broken E71–D80 link. Note that this link is stable though the whole simulation of mutant R64A. Thus, residues R64 in WT and Y82A proteins play the destabilizing role.

In both WT and Y82A proteins, arginine R64 can interact with D80, but these structures demonstrate different inactivation behavior. Our simulations indicate that the difference in the inactivation has a dynamical origin. Residue R64 moves faster and creates quicker a H-bond with D80 in mutant Y82A than in WT. The rate of H-bond creation depends on the conformation of the leucine in position 81 (L81). This rate primarily controls by the rotation of L81 side chain, which can open by *flipping*, when angle $\chi 1_{81}$ changes from $-63^{\circ }$ to $185^{\circ }$, or obstruct, when residue L81 is in that conformation found in the crystallographic structure, the path toward forming the D80–R64 H-bond (Figure 3a). Conformational changes of L81 have, therefore, a critical regulatory role in the dynamics of residue R64.

In turn, the dynamics of L81 are associated with additional readjustments in the amino acid sequence L81-X82-P83-V84, roughly definable as pivoting around the residue in position 82 (*X82-pivoting* (Figure 3a). Collective motions of this sequence can promote the flipping of L81 and a small drift of its backbone. The X82-pivoting is different in three considered proteins. The main differences are reflected in the RMSDs of residues surrounding residue 82 (X82) (Figure S5 in the Supplementary Materials). In all the three proteins, the RMSDs are similar for X82 (where X is tyrosine Y in the WT and R64A and alanine A in Y82A). In contrast, the RMSDs of the surrounding residues (L81, P83, and V84) correspond to the inactivation probabilities R64A < WT < Y82A, that is, the RMSDs are larger for Y82A and smaller for R64A than for WT. The lowest RMSDs for mutant R64A are due to the absence of a residue in position 64, which is capable of interacting with L81 via X82-pivoting. In WT and Y82A proteins, the R64–L81 interaction is controlled by bulky tyrosine Y82 and non-bulky alanine A82, respectively. In the WT protein, therefore, the motion of L81 is slower and more limited than in mutant Y82A, while in Y82A, the dynamics of L81 are faster and accompanied by a noticeable backbone drift (Figure 3a). Note that the described X82-pivoting can furthermore explain the conformational rearrangements of Y82 suggested in the experimental investigation of the C-type inactivation [17].

Thus, the mutation in position 82 changes the dynamics of residues close to the filter region, mainly affecting the conformation of L81. The enhancement of L81 transitions in the deep inactivating mutant Y82A causes the promotion of D80–R64 interactions because the dynamics of L81 and R64 are strongly coupled. Note that the X82-pivoting also alters the dynamics of residues V84 which can access D80 in a similar manner as R64. A comparable influence of V84 on D80, therefore, can be hypothesized. However, if such influence exists, it was masked by a stronger R64–D80 interaction.

For verifying the regulatory role of L81, mutant L81A (Figure 1) was additionally considered. The probability of the creation of the D80–R64 H-bond was compared for three proteins: WT, Y82A, and L81A. All the proteins were simulated with the same initial configuration, and the probability was calculated for the same time interval 10 ns. Consistently with the presented above results, the simulations confirm that the rate of the H-bond creation depends on residues L81, with a trend in the probability L81A > Y82A > WT (Figure 3b). The probability is larger in Y82A with respect to the WT by the enhanced fluctuations of L81. The probability becomes even larger in L81A when L81 is directly substituted by the small alanine which interferes less with R64 motion.

The results of this subsection show that proteins WT, R64A, and Y82A differ from each other in the dynamics of a few residues: primarily arginines R64 and R89, and leucine L81 that regulates the D80–R64 interaction. The cooperative dynamics of these three residues have a destabilizing effect on the triad E71-D80-W67 and, therefore, affect the pore region. Note that glutamate in position 71 (E71) has a non-trivial influence on the inactivation. In the absence of E71, mutant E71A is very flexible, but the inactivation is suppressed entirely. The presence of E71 is, therefore, essential to observe the inactivation, as E71 limits the flexibility of the pore region and strongly affects the motion of D80.

## 4. The Interactions of Residues and Ions in the WT Protein

### 4.1. Influence of Arginines R64 and R89 on D80, the SF and Ions

**Figure 4:**
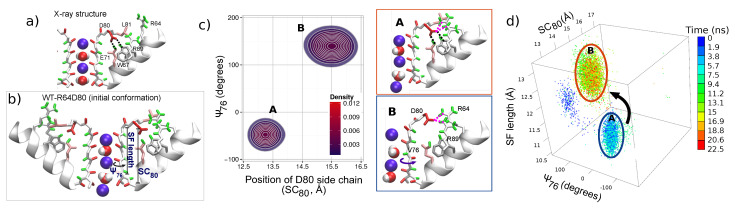
Panels (**a**,**b**) compare of the X-ray structure of the WT protein (a) and the initial configuration for simulation WT-R64D80 (b). Panel (**c**) shows the correlation between the states of D80 and the SF by means of the two-dimensional probability density for position SC${}_{80}$ and angle $\psi_{76}$ (see “Methods”) during first 22.5 ns of simulation WT-R64D80, i.e., before ion configuration KwK0K was reached. Two meta-stable states are denoted by letters **A** and **B**. Snapshots corresponding to each of the two states are shown on the right side panels. State **A** is the initial state in which E71-D80-W67 linkages were present. State **B** is characterized by an outer movement of D80 which followed by the break of E71-D80-W67 linkages and TVGYGD rearrangements. Panel (**d**) reports time evolution of subunit C in the conformational space defined by (i) $\psi_{76}$, (ii) SC${}_{80}$, and (iii) the length of the TVGYG sequence, SF length. The trajectory in the conformational space is coded by color scale shown in the colorbar. Letters **A** and **B** indicate the same states as in panel (c).

For understanding the action of arginines R64 and R89 in the WT protein, conformations with residue R64 close to residue D80 were investigated by a simulation started with a particular initial configuration. In order to enhance the probability of the R64-D80 interaction, an unbiased simulation (denoted *WT-R64D80*, duration of 45 ns) of the KcsA WT protein was commenced from a conformation with residues R64 were near D80 in all the four subunits. Details of how the initial configuration was obtained are given in the section “Methods”. Figure 4b shows the initial configuration characterized by the filter occupancy wKwKw+K; the flipped state of L81 and R64 is close to D80 in all subunits. Note that in subunit C, residue R64 forms a H-bond with D80 during a short equilibration in a preparation stage (see Figure S7 in the Supplementary Materials, starting point).

Two positively charged arginines R64 and R69 can exert a sufficiently strong combined upward force on negatively charged residue D80 to overcome the strong downward attraction toward E71. From the beginning of simulation WT-R64D80, this force resulted in a large mobility of the pore region. A long breakage of E71-D80-W67 linkages (for 17 ns) occurred in subunit C as well as brief disruptions of the linkages in other subunits were observed. Conformational rearrangements in residues and content (K${}^{+}$ ions and water molecules) of the SF accompanied these disruption events. Representative snapshots of changes in the SF are shown in the Supplementary Materials, Figures S7 and S8. The rearrangements observed in the subunit C were analyzed using three order parameters: Angle $\psi_{76}$, distance SC${}_{80}$ and the length of the TVGYG sequence of the subunit, SF length. Figure 4 shows the trajectory, which reflects the time evolution of the system, in the conformational space defined by these three order parameters for the first 22.5 ns of the simulation. Initially, several transitions of residues V76 (angle $\psi_{76}$ switches back and forth between $-50^{\circ }$ and $145^{\circ }$) were observed. These transitions demonstrate the inherent flexibility of the V76/G77 peptide group, which is sensitive to changes in the SF. The trajectory also shows that after 5 ns the E71-D80-W67 triad broke and residues D80 moved outward (distance SC${}_{80}$ changes from 13.5 Å to 15.5 Å). All these changes were promoted by residue R64. Residue D80 accommodated an upward state with H-bonds formed between either D80 and R64, or D80 and R89, or D80 and both arginines (see Figure S8 in Supplementary Materials). This upward state of D80 caused stretching of the TVGYGD sequence of the SF (SF length increases), and residue V76 switched to a meta-stable flipped state. The described changes correspond to the transition from the state **A** to the state **B** in the conformational space (Figure 2). The two-dimensional density for distance SC${}_{80}$ and angle $\psi_{76}$ shown in Figure 2b emphasizes a meta-stable character of the distorted state **B** and its dependence on the position of D80 side chain.

**Figure 5:**
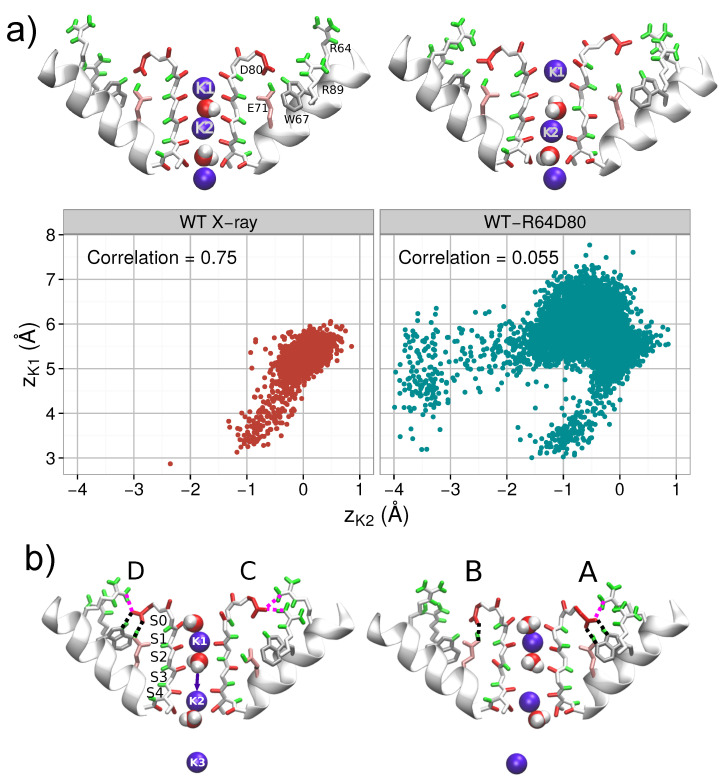
Panel (**a**) illustrates the correlation of the position of permeating K${}^{+}$ ions (K1 and K2) for two different conformations of WT: (i) the X-ray conformation in which R64 was far from D80 in all subunits; and (ii) the confirmation used in simulation WT-R64D80 with R64 near D80 in all the subunits. Only a part (durations of 7 ns for (i) and 15 ns for (ii), respectively) of the simulations with identical filter occupancy wKwKw+K were considered. For simulation WT-R64D80 the part corresponds to broken E71-D80-W67 H-bonds. Representative snapshots and *z*-positions of two ions in the SF on the state plane of $z_{K1}$ and $z_{K2}$ for each configuration are shown. Panel (**b**) depicts a configuration of the channel after the inward transition of the innermost ion K2 (wKwKw+K ⇄ wKw0K+K) occurred. All four subunits denoted by letters A–D are shown.

Figure 5 and Figure S9 in Supplementary Materials show significant consequences of the distortions in subunit C on the elements bound to the filter, and in particular, on the permeating K${}^{+}$ ions, which facilitate in spreading the distortions among the other three subunits. The changes in the permeation path can be characterized by the correlation between the positions of K${}^{+}$ ions in the SF. Let us stress that strongly correlated motion of ions was considered as being the fundamental feature of the knock-on mechanism of the permeation in previous works [92,93]. Simulations started from the X-ray configuration demonstrate the presence of such correlated dynamics of ions in the SF (see Figure 5a): Pearson’s coefficient is large (around 0.75) and the positions of ions K1 and K2 are linearly correlated. In the distorted state observed in simulation WT-R64D80 (state **B** in Figure 2b), the correlation between K${}^{+}$ ions is lost; Pearson’s coefficient is close to zero (Figure 5a). Ions in the SF become more flexible in the binding sites, that leads to weakening in the spatial definition of the K${}^{+}$ sites (Supplementary Materials, Figure S9). An unexpected transition of the innermost ion (K2) toward the intracellular side was observed (wKwKw+K ⇄ wKw0K+K, Figure 5b). This transition occurred in the reverse direction with respect to the permeation path. Note that such a transition was not observed in simulations started from the X-ray conformation of the WT protein. The observation of the inverse transition is particularly important because it reveals the influence of protein distortions on single K${}^{+}$ permeation events. This influence additionally can explain the different free-energy barriers obtained for the permeation path using biased approaches which induce distortions of some parts of the KcsA protein [58,86,90,94,95].

The described distorted state of the SF is observed during the initial part of simulation WT-R64D80. In the later stage, ions underwent several further rearrangements. One of the rearrangements is the ion (K3) from the cavity enters the SF (wKwKw+K ⟶ wKwKK) that leads to the re-establishment of E71-D80-W67 linkages. Then, the transition of the outermost ion (K1) to the site S0 (wKwKK ⟶ KwK0K) led to a configuration close to those observed in the conductive state of the X-ray structure. The latter result suggests that the conformation in which a K${}^{+}$ ion is bound to site S0 stabilizes the filter structure.

Thus, simulation WT-R64D80 demonstrates that conformational changes of the SF are dependent on a strongly correlated network of residues, in which aspartate D80 plays the central role. States of D80 with the broken E71-D80-W67 triad are promoted by the combined action of arginines R64 and R89. Furthermore, these states of D80 can destabilize the SF and cause filter’s distortions. The latter affects the dynamics of ions and can prevent the permeation of ions. In turn, ions permeation through the SF can either enhance or suppress the destabilization effect.

### 4.2. Energetics of the Arginine Motions

Simulation WT-R64D80 was started from a particular initial configuration, and the observed changes in the SF are transient. In this subsection, therefore, the energetics of the changes are studied using a technique called well-tempered metadynamics (wt-metaD). Wt-metaD (see Supplementary Materials for further details) is a theoretical method which belongs to the family of the biased methods and has been successfully applied for both to accelerate the observation of rare events and to reconstruct free energy surfaces (FES) [62,76].

The analysis of the dynamics of mutants and simulation WT-R64D80 demonstrate that the interactions of two arginines R64 and R89 with D80 can trigger rearrangements which change the shape of the channel pore, hence alter the ion permeation in KcsA. The dynamics of R64 is strongly coupled with leucine L81 which regulates the D80–R64 interaction. Therefore, the motion of R64 needs to be analyzed together with the motion of L81. Arginines R89 is not directly controlled by neighboring residues and can be studied alone. Energetics of the motion of R69 are described in the Supplementary Materials (section “Energetics of the arginine motions”).

**Figure 6:**
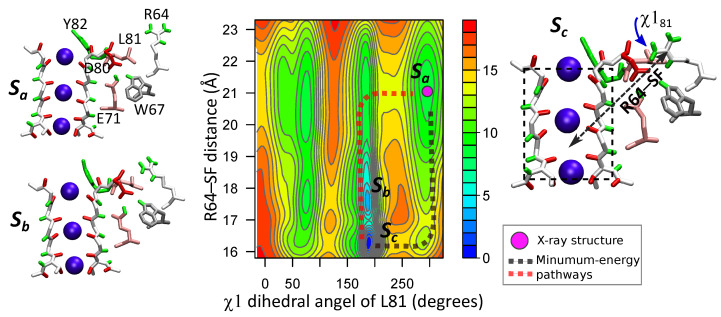
In the middle, two-dimensional FES computed via the wt-metaD approach is shown with respect to the distance between R64 and the SF (R64–SF) and angle $\chi 1_{81}$. In order to aid the visualization, angle $\chi 1_{81}$ is reported in the range (0,360), instead of the standard (−180,180). The FES is shown in kcal/mol, lines in the contour plot are drawn every 1 kcal/mol. Configurations of residues for three different multi-stable states: $S_{a}$, $S_{b}$ and $S_{c}$, are shown on the sides of the FES plot. These three states are denoted on the FES. State $S_{a}$ is with non-flipped residue L81 and close to the X-ray structure of KcsA. L81 is in the flipped conformation for states $S_{b}$ and $S_{c}$. Residue R64 forms a H-bound to D80 in the state $S_{c}$. The global minimum of the FES is state $S_{c}$.

For characterizing the dynamics of R64 and L81 the two-dimensional FES was calculated for the following order parameters: (i) the distance between R64 side chain and the center of mass of the SF (*R64–SF*), and (ii) angle $\chi 1_{81}$ (more details in section “Methods” and Supplementary Materials). The total sampling length of the wt-metaD simulation was 122 ns. The computed FES, shown in Figure 6, confirms the interplay between R64 and L81, and the regulatory role of the latter. There are several multi-stable states on the two-dimensional FES. State $S_{a}$ is with non-flipped residue L81 ($\chi 1_{81}\approx 297^{\circ }$) and residue R64 which is far from the SF (R64–SF >19 Å). This state is close to the X-ray structure of KcsA. It demonstrates that when L81 is in the non-flipped conformation, R64 tends to be away from the filter and D80. On the other hand, when L81 is in the flipped conformation ($\chi 1_{81}\approx 185^{\circ }$) residue R64 can approach closer to the SF (states $S_{b}$ and $S_{c}$, R64–SF <18 Å). Residues D80 and R64 form a H-bond in the state $S_{c}$. Two minimal-energy paths $S_{a}\to S_{c}$ are shown by dotted lines in the FES plane. The first path, highlighted by the magenta line, consists of an initial flipping of the L81 side chain ($\chi 1_{81}$ from ~$297^{\circ }$ to ~$185^{\circ }$) followed by the subsequent movement of R64 towards the SF along a downward gradient. The second path, highlighted by the black line, involves the creation of an initially relatively unstable D80–R64 H-bond which is lately stabilized by the flipping of the L81 side chain. Both paths have a similar energy barrier (5 kcal/mol).

Note that the energy barrier for the inverse transition $S_{c}\to S_{a}$ is significantly higher (13–15 kcal/mol) than for $S_{a}\to S_{c}$. It means that state $S_{c}$ corresponds to the global minimum of the FES and the configuration with a H-bound between R64 and D80 and with L81 in the flipped conformation should be observed in X-ray structural studies [28]. An analogous result, with the R89–D80 H-bond in the most probable state, was also obtained by free-energy calculations of the R89 motion for two out of three configurations of ions (see Supplementary Materials, section “Energetics of the arginine motions”).

Thus, the most probable positions of arginines R64 and R89 observed in the wt-metaD calculations are different from those in the X-ray structure [28]. These positions difference can be explained by interactions between the protein and surrounding lipids. In fact, numerous experiments indicate that in common with other K${}^{+}$ channels, KcsA channel is stabilized in the conductive state by the presence of the anionic lipids. In contrast, the channel is primarily non-conductive for the non-anionic lipids [20,33]. Deol et al. [96] revealed, by means of molecular dynamics simulations, that R64 and R89 can form strong, long-lived H-bonds with the head groups of the anionic lipids. Later, this result was experimentally confirmed [33]. This arginine–lipid interaction could bring the arginines in positions close to those determined by the X-ray experiment [28].

Our wt-metaD simulations were performed in the absence of anionic lipids, using neutral POPC lipids which as shown experimentally have no specific interaction with KcsA [33]. However, the radial distribution function that characterizes the interaction of Cl${}^{-}$ ions in the bulk with residues R64 and R89, confirms the strong affinity between the arginines and negatively charged species (see Figure S11 in the Supplementary Materials). Because of this affinity, the computed FESs (Figure 6 and Figure S10 in Supplementary Materials) show that the most stable position of R64 and R89 are located in proximity to the negatively charged D80. The presence of the anionic lipids would make this position less probable by additional interactions between the arginines and these lipids. Another factor affecting the arginines is locations of ions in the SF. For example, when ions occupy sites S0, S2 and S4, the probabilities of finding R89, respectively, in proximity to D80 and far from D80 are equal (see Figure S10 in Supplementary Materials). The influence of ions’ configuration on the dynamics of R64 is considered in the next subsection.

### 4.3. Opposite Influence of R64 and a K${}^{+}$ Ion Bound to S0 on the E71–D80 H-bond

**Figure 7:**
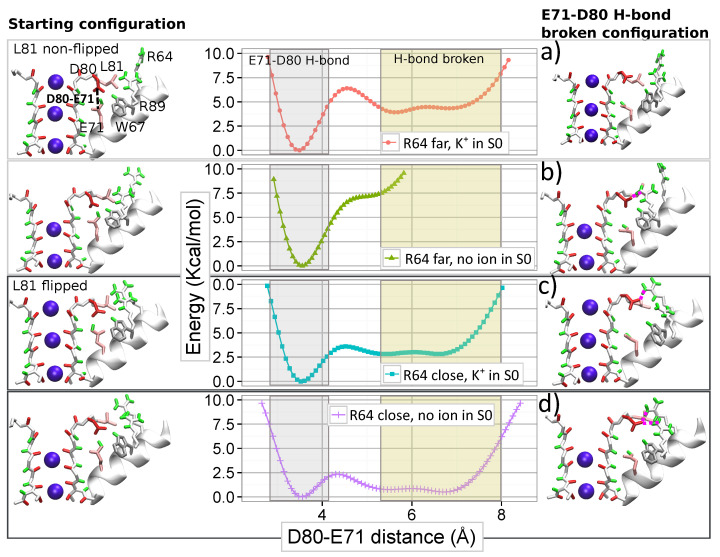
Graphs in the middle show the FESs for the distance between E71 and D80 in different cases: (**a**) R64 is far from D80 and ions configuration “KwK0K”, (**b**) R64 is far from D80 and ions configuration “wwK0K”, (**c**) R64 is close to D80 and ions configuration “KwK0K”, and (**d**) R64 is close to D80 and ions configuration “wwK0K”. A starting configuration for each wt-metaD simulation is shown on the left side of the figure. Examples of a configuration with a broken H-bond between E71 and D80 are shown on the right side of the figure for each corresponding initial configuration.

For characterizing the simultaneous action of arginine R64 and ions in the SF on the strong H-bond between E71 and D80, we calculated the FES for the interaction of E71 and D80 in different configurations of the SF. The distance between E71 and D80 residues is selected as the order parameter for the FES. The calculated FESs for arginine R89 (see Supplementary Materials, Figure S10) suggest that a K${}^{+}$ ion in site S0 stabilizes the E71–D80 H-bond by reducing the probability of the R89–D80 interaction. Free energy calculations were, therefore, performed for two ions conformations: one is “KwK0K” with an ion bound to S0, and the other is “wwK0K” without an ion in S0. Two different positions of R64 with respect to D80, near and far away respectively, were additionally considered. Thus, the FES were calculated for four different configurations of R64 and ions in the SF (see Figure 7). Further details of the FES calculations are reported in Supplementary Materials (Section 7). Note that configurations with a water molecule occupied site S3 (“KwKwK” and “wwKwK”) were also considered and the corresponding results are reported in Supplementary Materials (Figure S12). These results are consistent with those presented below.

The FESs in Figure 7 demonstrate the strong mutual influence between the filter occupancy and the position of R64 on the E71–D80 H-bond. The interaction between E71 and D80, therefore, does not merely depend on the nature of the residues and the nearby solvent molecules (water). Still, it originates from many different elements which constitute a strongly interacted (correlated) system.

If an ion is absent in site S0 and simultaneously R64 is far from D80 (Figure 7b), the E71–D80 H-bond is the only stable state in the FES. However, the proximity of R64 to D80 makes breaking the E71–D80 H-bond possible and leads to new meta-stable states without the bond (Figure 7d). The energetic barrier for the breaking the H-bond is relatively small (around 2.5 kcal/mol) and slightly higher (by 0.2 kcal/mol) than the barrier for re-establishing the H-bond. These new states without the interaction between E71 and D80 are close to those that led to distorted configurations in the SF observed during simulation WT-R64D80.

The presence of a K${}^{+}$ ion in site S0 changes the observed picture. In the case of R64 located far from D80, the presence of an ion leads to new states with the broken E71–D80 H-bond (compare Figure 7b and Figure 7a). However, the new states are less stable than the state with the H-bond, and the energetic barrier for the bond breaking is high (around 6.5 kcal/mol). When R64 is close to D80, the occupation of site S0 increases the energetic barrier for the breaking the H-bond and makes states without the H-bond significantly less stable (compare Figure 7d and Figure 7c). In this case, the barrier for the bond breaking is around 4 kcal/mol, and for the re-establishing, the barrier is four times less (Figure 7c). Thus, a K${}^{+}$ ion occupied site S0 opposes the destabilizing influence of arginine R64, favoring the presence of the E71-D80 H-bond. The absence of an ion in site S0 induces a widening of the site that facilitates the approach of R64 to D80 and destabilizing the E71–D80 H-bond.

These results demonstrate broad cooperation between residues and ions in controlling the dynamics of the pore region. Note that the described role of the occupation of site S0 by an ion provides a mechanistic and energetic insight to the hypothesis of a ‘foot-in-the-door’ mechanism, widely discussed in the literature for interpreting some experimental results [9,16,38]. In particular, the strong dependence of the current on the extracellular K${}^{+}$ concentration was observed experimentally [9,10,38,43,44]. For explaining this strong dependence, different authors have hypothesized that ion’s occupancy in the SF rises for the high concentration of ions and an ion resident in the filter stabilizes the conductive conformation. This hypothesis was supported further by the evidence that ions with a longer occupancy (Rb${}^{+}$, Cs${}^{+}$, and NH${}_{4}^{+}$) slow down the switching of the ion channel into the inactivated state [9,38]. According to our results, an ion in site S0 appears as the most valuable candidate for playing the role of the “foot-in-the-door”.

As previously mentioned, Cordero et al. [14] reported a flipped structure in mutant E71A, where the replacement of glutamate E71 by alanine A71 effectively remove the E71–D80 H-bond that leads to broad outward movement of D80 and large rearrangements in the V76 region. In all our simulations, the WT protein never adopted a similar configuration, even for states with considerable free energies. It implies that residue E71 consistently plays a dual role in shaping the WT ion channel through the strong electrostatic interaction between E71 and D80 and through a steric hindrance of large rearrangements in the region of V76.

## 5. Conclusions

In this work, a comparative analysis of the dynamics of the WT KcsA ion channel and mutants E71A, Y82A, R64A, and L81A was conducted using molecular dynamics simulations. This analysis helped us to identify a set of residues which control the state of the SF. The interactions between the identified residues and the interdependence between the residues and ions in the SF were characterized by free-energy calculations using well-tempered metadynamics [62]. A detailed description was provided for the residues which most prominently outlined the state of the SF and the influence of the ion permeation path. Our investigations revealed that the permeation path is regulated by a strongly interconnected dynamical system. The system is centered on aspartate D80, which is linked to neighboring H-bond donors, includes ions in the SF and residues located far from the SF. Key features of this interconnected system were described, that provides a consistent and unifying picture for some experimental results on the regulation of KcsA activities. These features are highlighted below.

First, the highly conserved aspartate D80 plays the critical role in changing the structure of the SF by translating broader dynamics of the protein to the filter structure because of the relatively rigid backbone of the conserved sequence GYGD of the SF. Thus, movements of residue D80 can trigger significant rearrangements of the whole pore.

Second, two arginines (R64 and R89) can strongly interact with D80 via H-bonding. This interaction facilitates movements of D80 that triggers the changes in the protein pore. While the D80–R89 interaction was previously described in the literature [91], the possibility of the D80–R64 H-bond and the destabilizing consequences of the combined action of these two arginines on D80 were described for the first time in this work. Between the two arginines, R64 was found to exert the strongest influence on D80, and thus on the ion flow.

Third, the local dynamics of the region behind the filter is regulated by conformational changes of leucine residue L81. These changes, in turn, are linked to collective motions of the amino acid sequence L81-Y82-P83-V84, in particular to a pivoting action on residue Y82. Additionally, the simulations provided the unambiguous evidence for the regulatory role of L81: the flipping of the L81 side chain facilitates the establishment of the D80–R64 H-bond.

Fourth, the destabilization effect of arginines R64 and R89 on states D80 is reduced by the presence of a K${}^{+}$ ion in the outermost binding site (S0) of the filter since the resulting electrostatic interactions stabilize the conductive structure.

We showed that the interactions between the two arginines (R64 and R89) and D80 induces the breaking the E71–D80 H-bond that could lead to a non-conducting state of the pore. This result provides an explanation of the necessity of the anionic lipids for observing the current in KcsA channel as the lipids can interact with both arginines [96], and this interaction reduces the probability of breaking the E71–D80 H-bond. Additionally, we showed that the occupancy of site S0 by an ion also stabilize the E71–D80 H-bond. The stabilizing influence of the ion bound to S0 offers an important insight into the “foot-in-the-door” mechanism proposed by various authors for explaining the influence of the extracellular K${}^{+}$ concentration in stabilizing the conductive state [9,38,43].

Our comparison between the dynamics of the WT protein and mutant E71A revealed a vital role of glutamate residue E71 in response to perturbations of the pore region. In the WT protein, the residue E71 participates in E71-D80-W67 linkages, which are considered as being an essential factor driving the filter toward non-conductive conformations [9,29]. Our results demonstrated that these linkages represent just a part of the more extensive strongly correlated network which dynamically and collectively participates in determining the state of the SF. The mutation of E71 with alanine in mutant E71A generated a non-inactivating pore with freely moving D80 [14]. We showed that in mutant E71A, residue D80 interacts with arginines R64 and R89. This interaction induces the strain on the SF, which adapts and relieves the perturbation through a flipping of V76 and a transition of D80 toward the extracellular (outer) region. As a result of this adaptation, the filter remains in a conducting state. The presence of E71 in the WT protein prevents such adaptation when D80 interacts with the two arginines. This interaction, therefore, leads to distorted configurations with complicated dynamics. The resulting complex picture is defined by ions and water molecules in the filter as well as by residues interacting or controlling the interaction between the arginines and D80.

**Figure 8:**
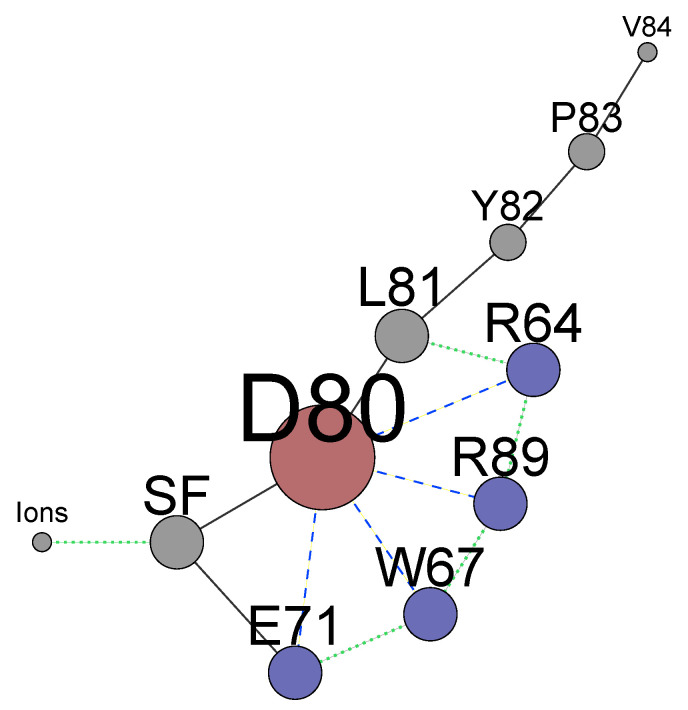
The network of residues that are determinant for the permeation path is drawn following certain rules: (i) blue-dashed lines represent non-bonded electrostatic interactions that can eventually lead to strong H-bonds; (ii) black lines represent connections through the backbones of the WT protein; and (iii) green dotted lines represent all the remaining non-bonded interactions, such as steric interactions or repulsions between positive or partially-positive charged groups. The sizes of the nodes are weighted according to the number of edges connecting each node. The label “SF” indicates the selectivity filter. The network was created using software package Gephi [99].

The summarized complex picture provided by this research can be represented as a network of weighted nodes which affect the permeation path (Figure 8). The sizes of the nodes are weighted according to the number of edges connecting each node. This figure reveals the primary importance of the residue D80, being the main hub. It forms the core of the network with the neighbouring H-bond donors E71, W67, and arginines R64 and R89 which mutual dynamical influence defines states of D80. The collaborative dynamics of the residues result either in the stabilization of the conductive conformation or in distorted states of the TVGYGD sequence of the SF. Note that the sequence belongs to the highly conserved signature sequence TXXTXGYGD observed in many potassium channels [16,29,31,97]. In these channels, the aspartate residue D, similar to D80 in KcsA, is surrounded by different H-bond donors. Thus, the existence of similar complex network might be a general feature in the regulation of the current in the K${}^{+}$ ion channels.

The significant mutual influence between the residues behind the SF and the ion occupancy in specific sites means that perturbations imposed on either residues or ions affect the KcsA channel’s state. It is reasonable to expect that numerous networks’ states have distinct permeation properties. Recent experiments [7,23] with modified phospholipids showed that the interaction of arginines R64 and R89 with added phosphatidic acid lipid enhances the conduction in the KcsA channel. These experiments confirm the results of Section 4, which describes the particular role of these arginines in regulating the network (Figure 8). In turn, the change of ion locations by an additional artificial force in biased MD simulations [34,58] could alter the residues behind the SF resulting in a non-conducting state with a high permeation barrier. It means that all the network components (Figure 8) should be included as collective variables in a biased MD simulation. Alternatively, a set of biased MD calculations for different states of the networks should be considered. The self-organized dynamics of the whole network define conducting or non-conducting states of the KcsA channel and considering the SF and ions only is not sufficient.

This dynamical network (Figure 8) is identified for the canonical structure 1KC4 with a closed inner gate. It is shown [35,36] that opening the inner gate leads to the perturbations on the backbones of the SF. Therefore, the inner gate should be included in this dynamical network as well. However, the possibility of an opposite influence of ions and residues near the SF on the inner gate is an open question. Several structures [34,52,54,59,60] with a conducting SF and an open inner gate were generated in silico by combining different crystallographic structures. Creating such structures should include a slow adaptation of the whole network to changes in the gate. The applied constraints on the SF backbones and ions only does not guarantee a realistic configuration of the SF. In this context, MD simulations of a transition of the inner gate from closed to open state are an essential missing link for clarifying the influence of the inner gate on the whole channel.

Current physical models (see, for example, the recent work in [98] and references therein) of the ion permeation in the KcsA channel consider a part of this network: ions and their interaction with the residues in the SF. Incorporating the whole network in physical models would lead to a more complex model, for example, the Markov state type, but a more realistic one. The representation of the protein’s complexity via this network would lead to a comprehensive description of complicated patterns of currents observed experimentally.

Results of Section 4.1 show that one of network’s states is non-conducting, and the channel in that state is inactive. This observation means that the inactivation can result from the dynamics of this network alone without the involvement of the gate residues. Future work will address the role of the network in the C-type inactivation.

## Data Availability

Data related to this research are openly available from the University of Warwick archive at https://wrap.warwick.ac.uk/143572.

## Supplementary Materials

The Supplementary Materials containing details of the methods, additional results and figures are available online at https://www.mdpi.com/1099-4300/23/1/72/s1.

## References

[1] Zhou M., Morais-Cabral J.H., Mann S., MacKinnon R. (2001). Potassium channel receptor site for the inactivation gate and quaternary amine inhibitors. Nature.

[2] Kratochvil H.T., Carr J.K., Matulef K., Annen A.W., Li H., Maj M., Ostmeyer J., Serrano A.L., Raghuraman H., Moran S.D. (2016). Instantaneous ion configurations in the K+ ion channel selectivity filter revealed by 2D IR spectroscopy. Science.

[3] Kopec W., Koepfer D.A., Vickery O.N., Bondarenko A.S., Jansen T.L.C., de Groot B.L., Zachariae U. (2018). Direct knock-on of desolvated ions governs strict ion selectivity in K+ channels. Nat. Chem..

[4] DeMarco K.R., Bekker S., Vorobyov I. (2019). Challenges and advances in atomistic simulations of potassium and sodium ion channel gating and permeation. J. Physiol..

[5] Sumikama T., Oiki S. (2019). Queueing arrival and release mechanism for K+ permeation through a potassium channel. J. Physiol. Sci..

[6] Xu Y., McDermott A.E. (2019). Inactivation in the potassium channel KcsA. J. Struct. Biol. X.

[7] Renart M., Giudici A., Diaz-Garcia C., Molina M., Morales A., Gonzalez-Ros J., Poveda J. (2020). Modulation of Function, Structure and Clustering of K+ Channels by Lipids: Lessons Learnt from KcsA. J. Mol. Sci..

[8] Strong S.E., Hestand N.J., Kananenka A.A., Zanni M.T., Skinner J.L. (2020). IR Spectroscopy Can Reveal the Mechanism of K+ Transport in Ion Channels. Biophys. J..

[9] Cordero-Morales J.F., Jogini V., Lewis A., Vasquez V., Cortes D.M., Roux B., Perozo E. (2007). Molecular driving forces determining potassium channel slow inactivation. Nat. Struct. Mol. Biol..

[10] Hoshi T., Armstrong C.M. (2013). C-type inactivation of voltage-gated K^+^ channels: Pore constriction or dilation?. J. Gen. Physiol..

[11] Cuello L.G., Cortes D.M., Perozo E. (2017). The gating cycle of a K+ channel at atomic resolution. Elife.

[12] Chakrapani S., Cordero-Morales J.F., Perozo E. (2007). A Quantitative Description of KcsA Gating I: Macroscopic Currents. J. Gen. Physiol..

[13] Chakrapani S., Cordero-Morales J.F., Perozo E. (2007). A Quantitative Description of KcsA Gating II: Single-Channel Currents. J. Gen. Physiol..

[14] Cordero-Morales J.F., Cuello L.G., Zhao Y., Jogini V., Cortes D.M., Roux B., Perozo E. (2006). Molecular determinants of gating at the potassium-channel selectivity filter. Nat. Struct. Mol. Biol..

[15] Cordero-Morales J.F., Cuello L.G., Perozo E. (2006). Voltage-dependent gating at the KcsA selectivity filter. Nat. Struct. Mol. Biol..

[16] Kurata H.T., Fedida D. (2006). A structural interpretation of voltage-gated potassium channel inactivation. Prog. Biophys. Mol. Biol..

[17] Raghuraman H., Islam S.M., Mukherjee S., Roux B., Perozo E. (2014). Dynamics transitions at the outer vestibule of the KcsA potassium channel during gating. Proc. Natl. Acad. Sci. USA.

[18] Lockless S.W., Zhou M., MacKinnon R. (2007). Structural and Thermodynamic Properties of Selective Ion Binding in a K+ Channel. PLoS Biol..

[19] Renart M.L., Montoya E., Fernández A.M., Molina M.L., Poveda J.A., Encinar J.A., Ayala J.L., Ferrer-Montiel A.V., Gómez J., Morales A. (2012). Contribution of Ion Binding Affinity to Ion Selectivity and Permeation in KcsA, a Model Potassium Channel. Biochemistry.

[20] Marius P., Zagnoni M., Sandison M.E., East J.M., Morgan H., Lee A.G. (2008). Binding of Anionic Lipids to at Least Three Nonannular Sites on the Potassium Channel KcsA is Required for Channel Opening. Biophys. J..

[21] Van der Cruijsen E.A.W., Nand D., Weingarth M., Prokofyev A., Hornig S., Cukkemane A.A., Bonvin A.M.J.J., Becker S., Hulse R.E., Perozo E. (2013). Importance of lipid–pore loop interface for potassium channel structure and function. Proc. Natl. Acad. Sci. USA.

[22] Radda R., Kim D., Nimigean C., Andersen O. (2014). Regulation of Ion Channel Function by the Host Lipid Bilayer Examined by a Stopped-Flow Spectrofluorometric Assay. Biophys. J..

[23] Poveda J., Giudici A.M., Renart M.L., Millet O., Morales A., Gonzalez-Ros J.M., Oakes V., Furini S., Domene C. (2019). Modulation of the potassium channel KcsA by anionic phospholipids: Role of arginines at the non-annular lipid binding sites. Biochim. Biophys. Acta (BBA) Biomembr..

[24] Hille B. (2001). Ion Channels of Excitable Membranes.

[25] Doyle D.A., Cabral J.M., Pfuetzner R.A., Kuo A., Gulbis J.M., Cohen S.L., Chait B.T., MacKinnon R. (1998). The Structure of the Potassium Channel: Molecular Basis of K^+^ Conduction and Selectivity. Science.

[26] Perozo E., Marien C.D., Cuello L.G. (1999). Structural Rearrangements Underlying K^+^-Channel Activation Gating. Science.

[27] Takeuchi K., Takahashi H., Kawano S., Shimada I. (2007). Identification and Characterization of the Slowly Exchanging pH-dependent Conformational Rearrangement in KcsA. J. Biol. Chem..

[28] Zhou Y., Morais-Cabral J.H., Kaufman A., MacKinnon R. (2001). Chemistry of ion coordination and hydration revealed by a K^+^ channel-Fab complex at 2.0 A resolution. Nature.

[29] Cordero-Morales J.F., Jogini V., Chakrapani S., Perozo E. (2011). A Multipoint Hydrogen-Bond Network Underlying KcsA C-Type Inactivation. Biophys. J..

[30] Chakrapani S., Cordero-Morales J.F., Jogini V., Pan A.C., Cortes D.M., Roux B., Perozo E. (2010). On the structural basis of modal gating behavior in K^+^ channels. Nat. Struct. Mol. Biol..

[31] Chapman M.L., Krovetz H.S., VanDongen A.M.J. (2001). GYGD pore motifs in neighbouring potassium channel subunits interact to determine ion selectivity. J. Physiol..

[32] Cheng W.W.L., McCoy J.G., Thompson A.N., Nichols C.G., Nimigean C.M. (2011). Mechanism for selectivity-inactivation coupling in KcsA potassium channels. Proc. Natl. Acad. Sci. USA.

[33] Marius P., de Planque M.R.R., Williamson P.T.F. (2012). Probing the interaction of lipids with the non-annular binding sites of the potassium channel KcsA by magic-angle spinning NMR. Biochim. Biophys. Acta (BBA) Biomembr..

[34] Florian T.H., David J.P., Wojciech W.N., Crina M.N., Berneche S. (2017). Mechanism of activation at the selectivity filter of the KcsA K+ channel. eLife.

[35] Labro A.J., Cortes D.M., Tilegenova C., Cuello L.G. (2018). Inverted allosteric coupling between activation and inactivation gates in K+ channels. Proc. Natl. Acad. Sci. USA.

[36] Sun Z., Xu Y., Zhang D., McDermott A.E. (2020). Probing allosteric coupling in a constitutively open mutant of the ion channel KcsA using solid-state NMR. Proc. Natl. Acad. Sci. USA.

[37] Devaraneni P.K., Komarov A.G., Costantino C.A., Devereaux J.J., Matulef K., Valiyaveetil F.I. (2013). Semisynthetic K+ channels show that the constricted conformation of the selectivity filter is not the C-type inactivated state. Proc. Natl. Acad. Sci. USA.

[38] Matulef K., Komarov A.G., Costantino C.A., Valiyaveetil F.I. (2013). Using protein backbone mutagenesis to dissect the link between ion occupancy and C-type inactivation in K+ channels. Proc. Natl. Acad. Sci. USA.

[39] Matulef K., Annen A., Nix J., Valiyaveetil F. (2016). Individual Ion Binding Sites in the K+ Channel Play Distinct Roles in C-type Inactivation and in Recovery from Inactivation. Structure.

[40] Baker K.A., Tzitzilonis C., Kwiatkowski W., Choe S., Riek R. (2007). Conformational dynamics of the KcsA potassium channel governs gating properties. Nat. Struct. Mol. Biol..

[41] Jekhmane S., Medeiros-Silva J., Li J., Kummerer F., Muller-Hermes C., Baldus M., Roux B., Weingarth M. (2019). Shifts in the selectivity filter dynamics cause modal gating in K+ channels. Nat. Commun..

[42] Li J., Ostmeyer J., Boulanger E., Rui H., Perozo E., Roux B. (2017). Chemical substitutions in the selectivity filter of potassium channels do not rule out constricted-like conformations for C-type inactivation. Proc. Natl. Acad. Sci. USA.

[43] Swenson R.P., Armstrong C.M. (1981). K+ channels close more slowly in the presence of external K+ and Rb+. Nature.

[44] Piasta K.N., Theobald D.L., Miller C. (2011). Potassium-selective block of barium permeation through single KcsA channels. J. Gen. Phys..

[45] Liu S., Focke P.J., Matulef K., Bian X., Moënne-Loccoz P., Valiyaveetil F.I., Lockless S.W. (2015). Ion-binding properties of a K+ channel selectivity filter in different conformations. Proc. Natl. Acad. Sci. USA.

[46] Renart M.L., Giudici A.M., Poveda J.A., Fedorov A., Berberan-Santos M.N., Prieto M., Diaz-Garcia C., Gonzalez-Ros J.M., Coutinho A. (2019). Conformational plasticity in the KcsA potassium channel pore helix revealed by homo-FRET studies. Sci. Rep..

[47] Sansom M.S., Shrivastava I.H., Bright J.N., Tate J., Capener C.E., Biggin P.C. (2002). Potassium channels: Structures, models, simulations. Biochim. Biophys. Acta (BBA) Biomembr..

[48] Maffeo C., Bhattacharya S., Yoo J., Wells D., Aksimentiev A. (2012). Modeling and Simulation of Ion Channels. Chem. Rev..

[49] Harpole T.J., Delemotte L. (2018). Conformational landscapes of membrane proteins delineated by enhanced sampling molecular dynamics simulations. Biochim. Biophys. Acta (BBA) Biomembr..

[50] Noskov S.Y., Bernèche S., Roux B. (2004). Control of ion selectivity in potassium channels by electrostatic and dynamic properties of carbonyl ligands. Nature.

[51] Tilegenova C., Cortes D.M., Jahovic N., Hardy E., Hariharan P., Guan L., Cuello L.G. (2019). Structure, function, and ion-binding properties of a K+ channel stabilized in the 2,4-ion–bound configuration. Proc. Natl. Acad. Sci. USA.

[52] Li J., Ostmeyer J., Cuello L.G., Perozo E., Roux B. (2018). Rapid constriction of the selectivity filter underlies C-type inactivation in the KcsA potassium channel. J. Gen. Physiol..

[53] Delemotte L. (2018). Opening leads to closing: Allosteric crosstalk between the activation and inactivation gates in KcsA. J. Gen. Physiol..

[54] Furini S., Domene C. (2020). Critical Assessment of Common Force Fields for Molecular Dynamics Simulations of Potassium Channels. J. Chem. Theory Comput..

[55] Cuello L.G., Jogini V., Cortes D.M., Pan A.C., Gagnon D.G., Dalmas O., Cordero-Morales J.F., Chakrapani S., Roux B., Perozo E. (2010). Structural basis for the coupling between activation and inactivation gates in K+ channels. Nature.

[56] Wojtas-Niziurski W., Meng Y., Roux B., Bernache S. (2013). Self-Learning Adaptive Umbrella Sampling Method for the Determination of Free Energy Landscapes in Multiple Dimensions. J. Chem. Theory Comput..

[57] LeMasurier M., Heginbotham L., Miller C. (2001). Kcsa: It’s a Potassium Channel. J. Gen. Physiol..

[58] Fowler P.W., Abad E., Beckstein O., Sansom M.S.P. (2013). Energetics of Multi-Ion Conduction Pathways in Potassium Ion Channels. J. Chem. Theory Comput..

[59] Jensen M.O., Jogini V., Eastwood M.P., Shaw D.E. (2013). Atomic-level simulation of current-voltage relationships in single-file ion channels. J. Gen. Physiol..

[60] Köpfer D.A., Song C., Gruene T., Sheldrick G.M., Zachariae U., de Groot B.L. (2014). Ion permeation in K+ channels occurs by direct Coulomb knock-on. Science.

[61] Sumikama T., Oiki S. (2016). Digitalized K+ Occupancy in the Nanocavity Holds and Releases Queues of K+ in a Channel. J. Am. Chem. Soc..

[62] Barducci A., Bussi G., Parrinello M. (2008). Well-Tempered Metadynamics: A Smoothly Converging and Tunable Free-Energy Method. Phys. Rev. Lett..

[63] Cosseddu S.M. (2013). Structure and Dynamics of Protein in the Permeation and Gating of Potassium Ion Channels: Identifying Molecular Determinants and Developing Coarse-Grained Approaches. Ph.D. Thesis.

[64] Phillips J.C., Braun R., Wang W., Gumbart J., Tajkhorshid E., Villa E., Chipot C., Skeel R.D., Kalé L., Schulten K. (2005). Scalable molecular dynamics with NAMD. J. Comput. Chem..

[65] Grubmüller H., Heller H., Windemuth A., Schulten K. (1991). Generalized Verlet Algorithm for Efficient Molecular Dynamics Simulations with Long-range Interactions. Mol. Simul..

[66] Tuckerman M., Berne B.J., Martyna G.J. (1992). Reversible multiple time scale molecular dynamics. J. Chem. Phys..

[67] Essmann U., Perera L., Berkowitz M.L., Darden T., Lee H., Pedersen L.G. (1995). A smooth particle mesh Ewald method. J. Chem. Phys..

[68] MacKerell A.D., Wiorkiewicz-Kuczera J., Karplus M. (1995). An all-atom empirical energy function for the simulation of nucleic acids. J. Am. Chem. Soc..

[69] Buck M., Bouguet-Bonnet S., Pastor R.W., MacKerell A.D. (2006). Importance of the CMAP Correction to the CHARMM22 Protein Force Field: Dynamics of Hen Lysozyme. Biophys. J..

[70] Klauda J.B., Venable R.M., Freites J.A., O’Connor J.W., Tobias D.J., Mondragon-Ramirez C., Vorobyov I., MacKerell A.D., Pastor R.W. (2010). Update of the CHARMM All-Atom Additive Force Field for Lipids: Validation on Six Lipid Types. J. Phys. Chem. B.

[71] Bernèche S., Roux B. (2002). The Ionization State and the Conformation of Glu-71 in the KcsA K^+^ Channel. Biophys. J..

[72] Bucher D., Guidoni L., Rothlisberger U. (2007). The Protonation State of the Glu-71/Asp-80 Residues in the KcsA Potassium Channel: A First-Principles QM/MM Molecular Dynamics Study. Biophys. J..

[73] Grubmüller H., Groll V. Solvate. https://www.mpibpc.mpg.de/grubmueller/solvate.

[74] Zhang L., Hermans J. (1996). Hydrophilicity of cavities in proteins. Proteins Struct. Funct. Bioinform..

[75] Oostenbrink C., Villa A., Mark A.E., Van Gunsteren W.F. (2004). A biomolecular force field based on the free enthalpy of hydration and solvation: The GROMOS force-field parameter sets 53A5 and 53A6. J. Comput. Chem..

[76] Laio A., Parrinello M. (2002). Escaping free-energy minima. Proc. Natl. Acad. Sci. USA.

[77] Fiorin G., Klein M.L., Hénin J. (2013). Using collective variables to drive molecular dynamics simulations. Mol. Phys..

[78] Humphrey W. (1996). VMD: Visual molecular dynamics. J. Mol. Graph..

[79] R Core Team (2013). R: A Language and Environment for Statistical Computing.

[80] Grant B.J., Rodrigues A.P.C., ElSawy K.M., McCammon J.A., Caves L.S.D. (2006). Bio3d: An R package for the comparative analysis of protein structures. Bioinformatics.

[81] Wickham H. (2009). ggplot2: Elegant Graphics for Data Analysis.

[82] Adler D., Murdoch D. rgl: 3D Visualization Device System (OpenGL).

[83] Fox J., Weisberg S. (2011). An R Companion to Applied Regression.

[84] Venables W.N., Ripley B.D. (2002). Modern Applied Statistics with S.

[85] Furrer R., Nychka D., Sain S. Fields: Tools for Spatial Data.

[86] Berneche S., Roux B. (2001). Energetics of ion conduction through the K^+^ channel. Nature.

[87] Bernèche S., Roux B. (2000). Molecular Dynamics of the KcsA K^+^ Channel in a Bilayer Membrane. Biophys. J..

[88] Bernèche S., Roux B. (2005). A Gate in the Selectivity Filter of Potassium Channels. Structure.

[89] Domene C., Klein M.L., Branduardi D., Gervasio F.L., Parrinello M. (2008). Conformational Changes and Gating at the Selectivity Filter of Potassium Channels. J. Am. Chem. Soc..

[90] Piccinini E., Ceccarelli M., Affinito F., Brunetti R., Jacoboni C. (2008). Biased Molecular Simulations for Free-Energy Mapping: A Comparison on the KcsA Channel as a Test Case. J. Chem. Theory Comput..

[91] Miloshevsky G.V., Jordan P.C. (2008). Conformational Changes in the Selectivity Filter of the Open-State KcsA Channel: An Energy Minimization Study. Biophys. J..

[92] Shrivastava I.H., Sansom M.S.P. (2000). Simulations of Ion Permeation Through a Potassium Channel: Molecular Dynamics of KcsA in a Phospholipid Bilayer. Biophys. J..

[93] Compoint M., Carloni P., Ramseyer C., Girardet C. (2004). Molecular dynamics study of the KcsA channel at 2.0-A resolution: Stability and concerted motions within the pore. Biochim. Biophys. Acta Biomem..

[94] Kim I., Allen T.W. (2011). On the selective ion binding hypothesis for potassium channels. Proc. Natl. Acad. Sci. USA.

[95] Furini S., Domene C. (2009). Atypical mechanism of conduction in potassium channels. Proc. Natl. Acad. Sci. USA.

[96] Deol S.S., Domene C., Bond P.J., Sansom M.S. (2006). Anionic Phospholipid Interactions with the Potassium Channel KcsA: Simulation Studies. Biophys. J..

[97] Catterall W.A. (2010). Ion channel voltage sensors: Structure, function, and pathophysiology. Neuron.

[98] Gibby W.A.T., Barabash M.L., Guardiani C., Luchinsky D.G., McClintock P.V.E. (2020). Physics of selective conduction and point mutation in biological ion channels. arXiv.

[99] Bastian M., Heymann S., Jacomy M. Gephi: An Open Source Software for Exploring and Manipulating Networks. Proceedings of the International AAAI Conference on Weblogs and Social Media.

