# Supplementary material for: Unraveling of a Strongly Correlated Dynamical Network of Residues Controlling the Permeation of Potassium in KcsA Ion Channel
Source: Entropy (Basel). 2021 Jan 6;23(1):72. doi: 10.3390/e23010072 (PMC7825352; doi:10.3390/e23010072)
Supplement: Supplementary file 1 [file entropy-23-00072-s001.pdf]

Supplementary Materials: Unraveling of a Strongly Correlated Dynamical Network of Residues Controlling the Permeation of Potassium in KcsA Ion Channel

Salvatore M. Cosseddu, Eunju Julia Choe and Igor A. Khovanov

1. Methods

The simulations were performed using NAMD 2.8 and NAMD 2.9[1] in the NpT ensemble with pressure 1.01 bar (Langevin piston implemented via Nosé-Hoover method[1]), temperature 310 K (Langevin thermostat[1]) and electrostatic forces were computed using the smooth particle-mesh Ewald method (SPME).[2] Metadynamics calculations were performed using the built-in *colvar* module.[3] A multiple-timestep algorithm was used[4,5] and different integration steps according to the scope of simulations:

- 1 fs for unbiased simulations, bonded interactions computed every time step, non-bonded non-electrostatic forces computed every 2 time steps and electrostatic forces every 4 time steps;
- 2 fs for biased simulations, bonded and non-bonded non-electrostatic forces computed every time step and electrostatic forces every 3 time steps;

Using longer integration steps (2 fs) is a common choice for biased simulations, since it allows longer sampling. The CHARMM27 force field (FF) was used for the protein, with a modification in the Lennard-Jones term to represent the interaction between K^+ and the carbonyl oxygens of the protein, CHARMM36 for the lipids and TIP3P for water.[6–8]

The system was prepared by embedding the X-ray structure (pdb code 1K4C, 6284 atoms; solved at 2 Å resolution[9]) into a membrane patch of 1-palmitoyl-2-oleoylphosphatidylcholine (POPC, 222 molecules and 29748 atoms) prepared using the VMD 1.8.6 membrane plug-in, modified in order to work with CHARMM36. The protein was solvated using the *Solvate* program by Grubmüller [10], and the VMD solvate plug-in (17740 water molecules, 53220 atoms). MD free energy sampling, and QM/MM calculations (quantum mechanical/molecular mechanical) suggest that, in the X-ray conformation, the E71/D80 pair is linked by a hydrogen bond (H-bond) where one residue is ionized, and the proton exchanges with a preference for E71.[11,12] Consistent with conventional MD force fields, this has been mimicked in this work by assigning the proton to E71 in every protein where the E71-D80 bridge is present (WT, R64A, Y82A, L81A). Water molecules inside the cavity that were not resolved in the X-ray structure were inserted using the DOWSER program.[13] The missing parameters for K^+ necessary for DOWSER were taken from the GROMOS87 FF for consistency.[14] The potassium concentration in the aqueous phase was 0.2 M, equal to the bulk concentration used in many experiments:[9,15–19] 75 Cl^- ions and 63 K^+ ions, with 2 K^+ in the selectivity filter and 1 K^+ in the cavity. The final system, showed in Fig. S1, consisted of 89390 atoms and a size of 95 $\times$ 95 $\times$ 100 Å.

Mutants were created in-silico from the crystal structure of the WT which allow more accurate comparisons, following a commonly-used approach for studying mutants features.[15,16,20] Relaxation of the WT and preparation of the mutants were performed in different steps before the production runs. (i) A 2 ns MD simulation in which the protein, water, ions and lipid head groups were frozen; this ensured melting of the lipid tails. (ii) A further 2 ns MD simulation in which the protein (only) was restrained with harmonic tethers. After this step, the membrane having relaxed around the protein, the in-silico mutants were created using the program psfgen[1] based on the CHARMM topologies.[7,21] A further 1 ns MD simulation in which all atoms were unrestrained was performed for each protein.

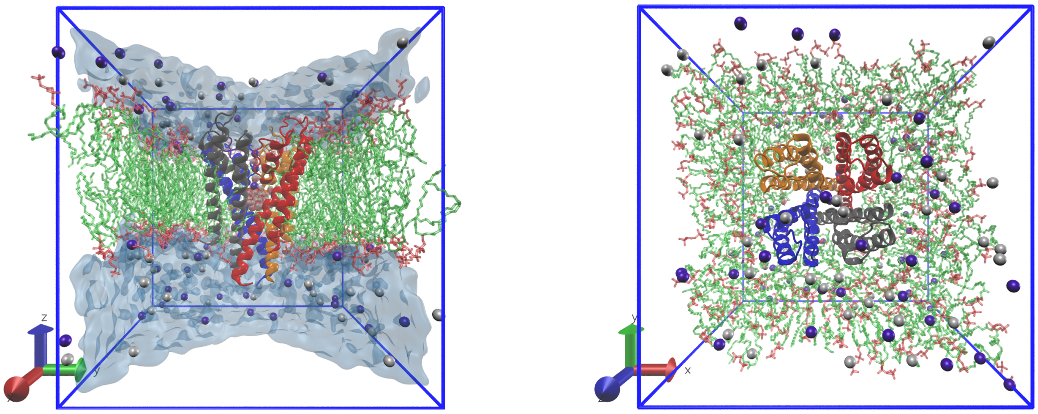

(a) Front view. **(b)** Top view.

Figure S1. The simulated protein, prepared from the X-ray structure of KcsA solved at 2 Å resolution embedded in a membrane patch of 1-palmitoyl-2-oleoylphosphatidylcholine (POPC), and immersed in solution 0.2 M of KCl. The different sizes of the atoms in the figure are chosen only for visualization purposes. The colors are chosen as follows: i) a different color is used for each protein domain; ii) lipid tails are in green; iii) lipid heads in red; iv) K^+ and Cl^- ions are in purple and gray, respectively. In the panel (**a**) the water molecules in the cavity are colored: red and white the oxygen and hydrogen atoms, respectively.

1.1. Well-tempered Metadynamics

Metadynamics (metaD) was originally developed as a flexible tool to explore new reaction pathways and accelerate the observation of rare events, as well as to reconstruct free energies.[22] It is based on a history-dependent potential added to the Hamiltonian of the system which biases the simulation toward sampling unexplored configurations. The bias potential is created as a sum of Gaussians centered along the trajectories projected onto a subset of degrees of freedom, usually referred to as collective variables (*CVs*). In the case of a comprehensive sampling, the detailed knowledge of the bias potential allows the unbiased Boltzmann distribution along the CVs to be retrieved, and thus allows calculation of the free energy hyper-surface.[23,24]

In the well-tempered metadynamics (wt-metaD), the height of a Gaussians added is history-dependent, and this dependence is associated with a parameter having the dimension of a temperature ΔT.

Dynamics of L81/R64 were investigated using $\Delta T = 1200$ K, the E71-D80 distance using $\Delta T =$ 1500 K. It is worth noting that, although important from a practical perspective, the computed FES will eventually converge to the real one irrespective of the particular choice of ΔT, for proper sampling.[24]

The initial deposition rate $\omega = w_0/\tau_G$ of the Gaussians affects relaxation over the orthogonal degrees of freedom.[24] The parameter w_0 appeared to be very important for the success of calculations and was chosen to be small (0.005 kcal/mol) in order to produce a smoother (quasi-adiabatic) filling of the biasing potential over the whole calculation, which is desirable to avoid the occurrence of non-physical events. The deposition time, τ_G, was selected to be 200 fs and a few preliminary tests suggested that the final result is not sensitive to the choice of this parameter in the cases under investigation.

2. Permeation in the V76 flipped configurations of E71A and WT

The flipping of V76 has been reported in several published works[25–29] and it has been suggested as being able to generate non-conductive conformations responsible for the inactivation of the filter (C-type inactivation) or for the modal-gating (flicker mode).[26,29] Any conformation in which the G77-V76 peptide group is rotated with respect to the putative conductive X-ray structure has been

referred to as "V76 flipped". The conductivity of the V76 flipped conformation for WT and E71A was tested by means of MD simulations to confirm this hypothesis.

Simulations were initiated from the last configurations obtained from simulations of WT and E71A in which V76 flipped appeared as meta-stable states with lifetime longer than 10 ns. Outward transitions of K^+ were induced by interchanging the positions of a K^+ ion from the bulk and a water molecule in the cavity. In this way, because of the closed state of inner gate which results in the cavity to be closed, the electrostatic repulsion of the two ions in the cavity led to promotion of the multi-ionic movements through the selectivity filter, associated with the permeation. Simulations were preceded by 10^5 steps of minimization (conjugate gradient and line search algorithm as implemented in NAMD[1]). Two simulations were performed for the WT with different initial velocities and one for E71A.

Both E71A and WT proved to be conductive and the flipped state of V76 to be reversible within a few ns when ion permeation occurs. It is likely that the creation of V76 flipped states, with longer lifetimes necessary to hinder K^+ current, with the longer lifetime being dependent on conformational changes in residues in the region behind the selectivity filter. Occurrence of a vacancy in the filter were observed during the permeation.

3. E71A dynamics

3.1. Comparison of D80 fluctuations between E71A and WT

Fig. S2 reports time series of the position of D80 side chain (SC_{80}) for WT and E71A proteins. The time series in Fig. S2 demonstrate the larger fluctuations of D80 in E71A than in WT. The standard deviation for the D80 position in E71A, 0.86 Å, was significantly bigger than in the WT, 0.22 Å (data used from all the four subunits). Notably, the value $SC_{80} \approx 13.5$ Å, which was very close to the value obtained for X-ray structure of the WT (≈ 13.3 Å), was found to be a metastable state for E71A, though completely stable (during the simulation) for the WT. This suggests the conformation of D80 found in the crystal structure is stabilized by the E71–D80 H-bond.

3.2. Time series for the transitions of E71A

Time series of the order parameters are reported in Fig S3 for the first 10 ns of the simulation (total length 24 ns) of E71A mutant. The order parameters are the following: i) the V76 ψ dihedral angle ψ_{76}, ii) the position SC_{80} of the D80 side chain (the distance between D80 C_γ and A73 C_α atoms); iii) the distance D80-R89 between D80 (C_γ atom) and the nearest R89 side chain (C_ζ) iv) the ψ dihedral angle of D80 ψ_{80}; v) the z coordinates of the outermost ion (K1) bound to the selectivity filter, centered relative to the COM of the selectivity filter. Rapid fluctuations in the instantaneous values of the parameters obscured trends in some cases, especially for the D80 position SC_{80}. So, only the smoothed trajectories are shown (moving averages procedure were calculated with the time window interval of 0.25 ns). The part of the simulation before the flipping of V76 (the first 1 ns have been omitted as relaxation) was subdivided into three sections : i) *before* the creation of D80–R89 H-bond, up to 2.5 ns (blue shadow area); ii) *during* the creation of D80–R89 H-bond, between 2.5 and 5.9 ns; and iii) *after* the creation of D80–R89 H-bond and before flipping of V76, between 5.9 and 9 ns (green shadow area). The time series show that the strengthening of the H-bond (D80-R89 distance reduces) is linked to D80 moving upward (SC_{80} increases). Note that the change in the position SC_{80} is more visible in Fig. S4 (discussed below). The creation of the H-bond is accompanied by the partial flipping of V76 (6.8 ns, a rotation by 50 degrees of ψ_{76}), soon followed (7 ns) by an inward movement of K1 (1.9 Å) to the position between sites S1 and S2, accompanied by an adjustment of the water molecule in S2 (not shown). At 9 ns the complete flipping of V76 was observed (red vertical line on the graphs in Fig. S3). Substituting the oxygen of the V76 backbone, this water molecule in site S2 re-established the square anti-prism geometry of the coordination sphere of K1, which then moved back to its original position in site S1. The final transition involved the D80 side chain (9.5 ns, ψ_{80} changes from -43 to $-22°$) that

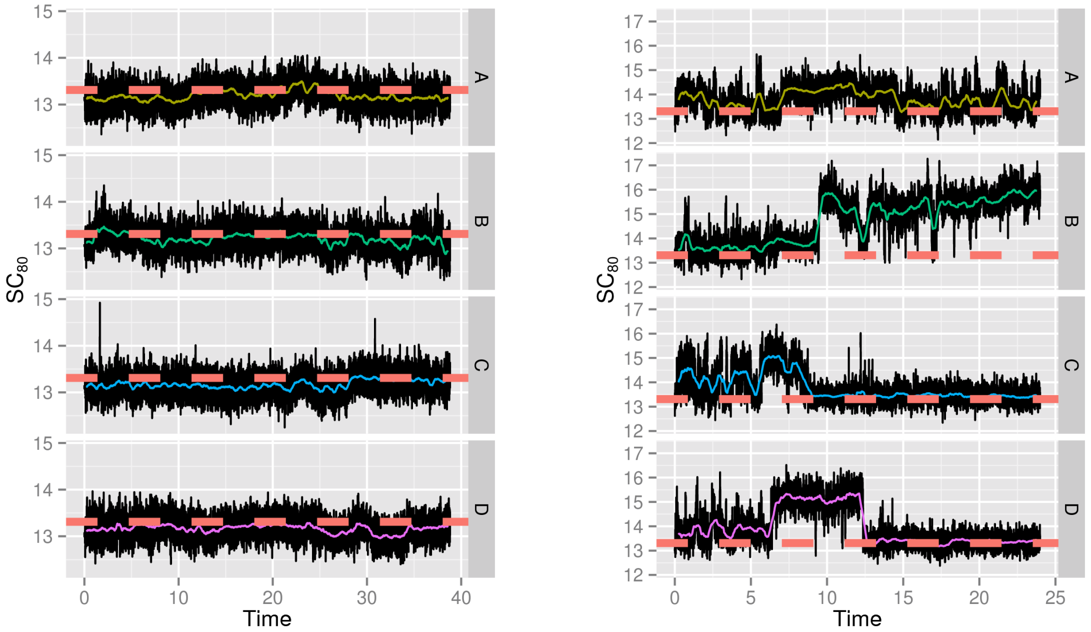

(a) Time series of D80 position for WT.

(b) Time series of D80 position for E71A.

Figure S2. Time series (reported in ns) of the position of D80 side chain in WT and E71A (SC_{80}, measured in Å). Black lines show the position stored every 2 ps, while colored lines show the smoothed trajectories (moving averages procedure calculated with the time window interval of 77 ps). Thick dashed lines indicate the value of d73CA in the crystal structure (pdb code 1K4C[9]).

broke the H-bond and restored the initial uncorrelated motions of the D80 and R89 side chains (see Figure 2 in the main text).

Fig. S4 shows the change in position of D80 following the creation of D80-R64 H-bond in the region *before* (blue) and *after* (light green). The mean value of the D80 position changes from 13.5 to 13.8 Å and two-sample Kolmogorov-Smirnov test indicates this difference is statistically significant with p-value $< 2.2 \cdot 10^{-16}$.

4. Comparison of the dynamics of residues

Fig. S5 reports the root mean square displacement (RMSD) of residues with respect to the reference X-ray crystal structure of WT. RMSDs computed for the simulations of WT, Y82A and R64A proteins. RMSD was estimated as time averaged deviation of the backbone atoms of a residue from the reference structure. Only the region surrounding the selectivity filter is considered (residues from 63 to 90). Note, the error bars in the figure are standard deviations of the RMSDs.

Fig. S6 shows an example of a partial rotation of the D80 side chain around the $\chi 1$ dihedral angle. The rotation was observed several times during the simulation of Y82A mutant.

5. Simulation WT-R64D80

In the present section, additional material is reported for the illustration of changes in the pore region during the simulation commenced with residue R64 near D80 in all the four subunits (simulation WT-R64D80). Fig. S7 illustrate conformational changes of the protein during the first part of the simulation. Fig. S8 shows snapshots of simulation WT-R64D80 when H-bonds between D80 and R64, or R89 or both R64 and R89 residues were formed. The probability density of z-coordinate of ions K1 and K2 are shown in Fig. S9 for simulations starting with WT-R64D80 and WT X-ray configurations.

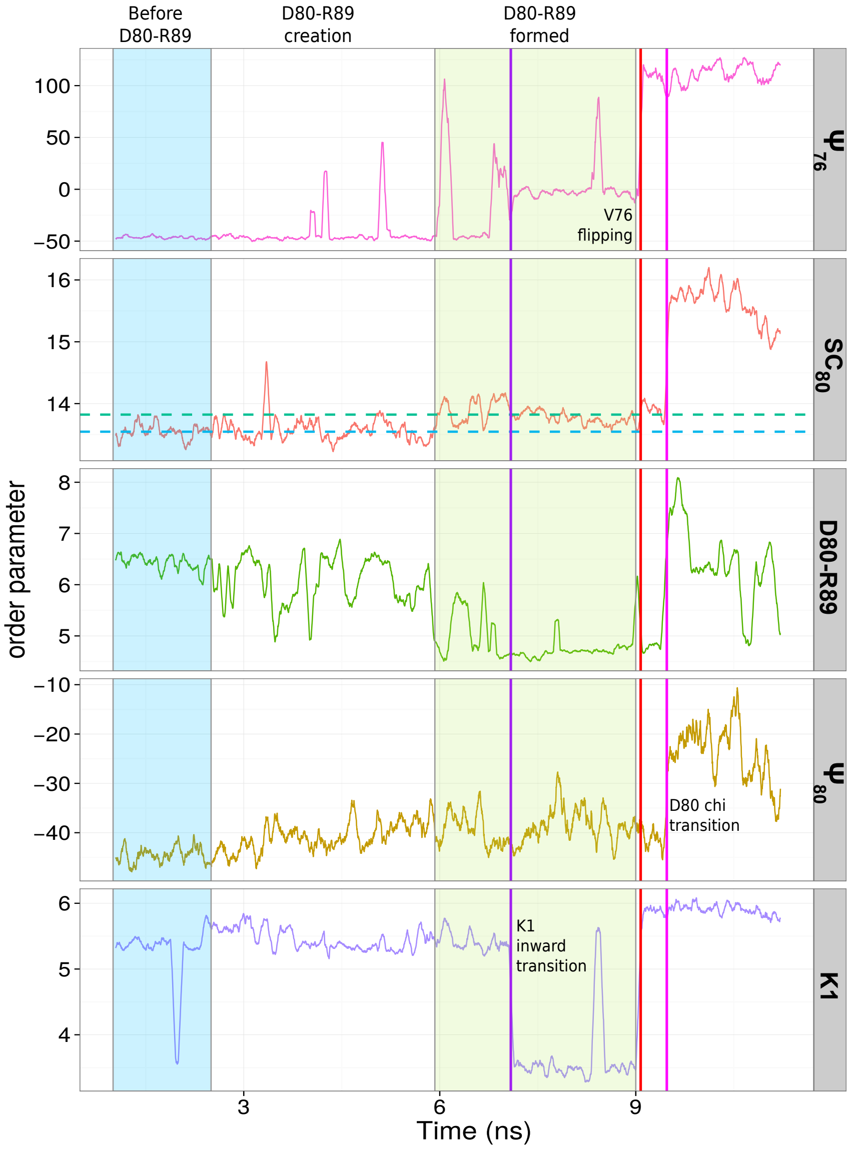

Figure S3. Time series of the order parameters illustrate the influence of the dynamics of D80 and R89 on the selectivity filter and permeating ions in E71A. Time series for subunit B are reported only. The colored lines represent the smoothed trajectories (moving average procedure applied, with the window interval of 0.25 ns long). The units for the D80 position, SC_{80}, D80-R89 distance and coordinate K1 are Å, the units for ψ_{76} and ψ_{80} are degrees. The reported sequence of changes in the trajectories demonstrates that the creation of D80–R89 H-bond can generate a stress on the backbone of the TVGYGD sequence that results in small conformational rearrangements. These backbone rearrangements are shown only for the residues V76 (initially a partial flipping, followed by the complete flipping) and for residue D80. The latter caused the breaking of D80–R89 H-bond, removing the stress on the filter structure.

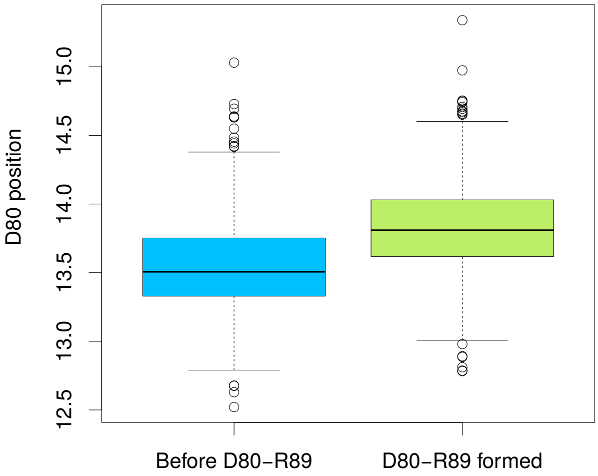

Figure S4. Boxplots of D80 position in E71A before (between 1 and 3.5 ns) and after (between 6.5 to 9 ns) the formation of the D80–R89 bond (see Fig. S3 for the time intervals).

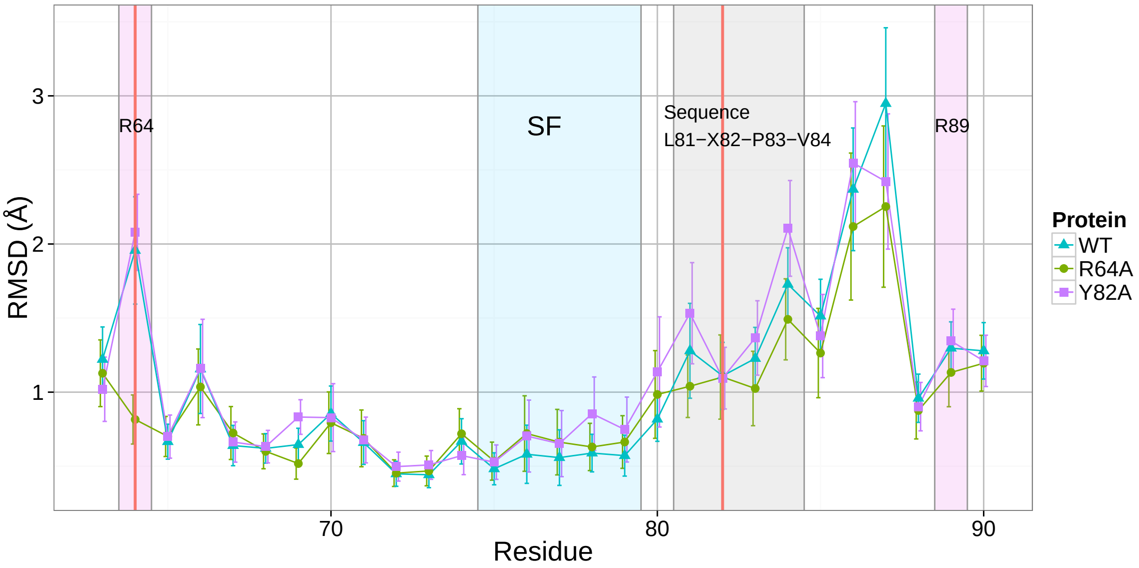

Figure S5. Root mean square displacement (RMSD) of residues in WT KcsA protein and its mutants R64A and Y82A are shown. The light blue shadowed area marks the selectivity filter (TVGYG sequence). The magenta lines and violet shadowed areas highlight the positions of the mutations in Y82A and R64A. The gray shadowed area highlights residues surrounding X82 (L81, P83, V84). These residues are associated with collective motions that are pivoting around X82.

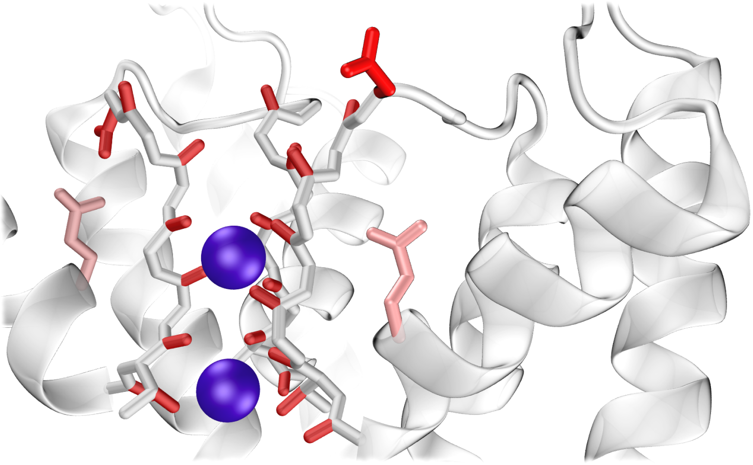

Figure S6. An example of the confirmation with a partial rotation of the D80 side chain around the $\chi 1$ dihedral angle in the simulation of Y82A is shown. Residue D80 is shown by red color.

6. Energetics of the arginine motions

6.1. Details of the FES calculation of R64/L81 motions

In the main text, the well-tempered metadynamics calculation of energetics of R64/L81 motions is presented. The two-dimensional FES was computed as functions of the CVs: i) the distance of the R64 side chain (C_ζ atom) from the COM of the selectivity filter (*R64–SF*); ii) the $\chi 1$ dihedral angle of the L81 residue ($\chi 1_{81}$). The calculation was obtained by considering only the configuration KwK0K of the K^+ in the pore, with a vacancy in S3. This configuration remained stable for the whole calculation and the FES shows a quick convergence.

6.2. Free energies for R89 motions

The FESs (Fig. S10) of the interaction between R69 and D80 was computed as a function of the distance, $SF - R89$, between the C_ζ atom of the R89 guanidino group and the COM of the selectivity filter. The FES was obtained on the base of two different unbiased simulations starting with the WT X-ray structure and using data from all the four subunits. The total sampling interval is 224 ns.

Initial estimations of the FES revealed that the FES depends on a configuration of ions in the selectivity filter. This observation confirms the existence of the strong link between the position of the K^+ ions in the selectivity filter and the state of the surrounding region. From all possible, three different configurations were typical and, therefore, the FES was calculated for them. These configurations are S2/S4, S1/S3, and S0/S2/S4, and the corresponding FESs are shown in Fig. S10.

The FESs confirm that in a similar manner to R64, the side chain of R89 can create a H-bond with D80. Two minimums corresponding to the presence and absence of the D80–R89 H-bond are clearly recognizable in the FESs, respectively at $SF - R89 \approx 15.7$ Å and $SF - R89 \approx 17.1$ Å. The barrier for the bond formation is defined by the transition from the state with $SF - R89 \approx 17.1$ Å to the state with $SF - R89 \approx 15.7$ Å. This barrier depends on the ions configuration and it is relatively low between 0.25 and 0.7 kcal/mol. The opposite barrier for breaking the D80–R89 H-bond depends on the ion configurations as well. The barriers are comparable for configuration S0/S2/S4, but the bond breaking is less probable when the outermost sites are not occupied. Thus, the R89–D80 H-bond is more stable when an ion is not in S0. Such a configuration is observed in the case of a low concentration of K^+ in the bulk, which is the condition when the inactivation probability is large.

The dependence of the FESs on the ion occupancy further confirms the role of K^+ ions bound to the filter in the dynamics of the pore region. However, the low barriers between the states in the FESs also indicate that the destabilization effect of R89 on E71-D80-W67 linkages is weaker in comparison to

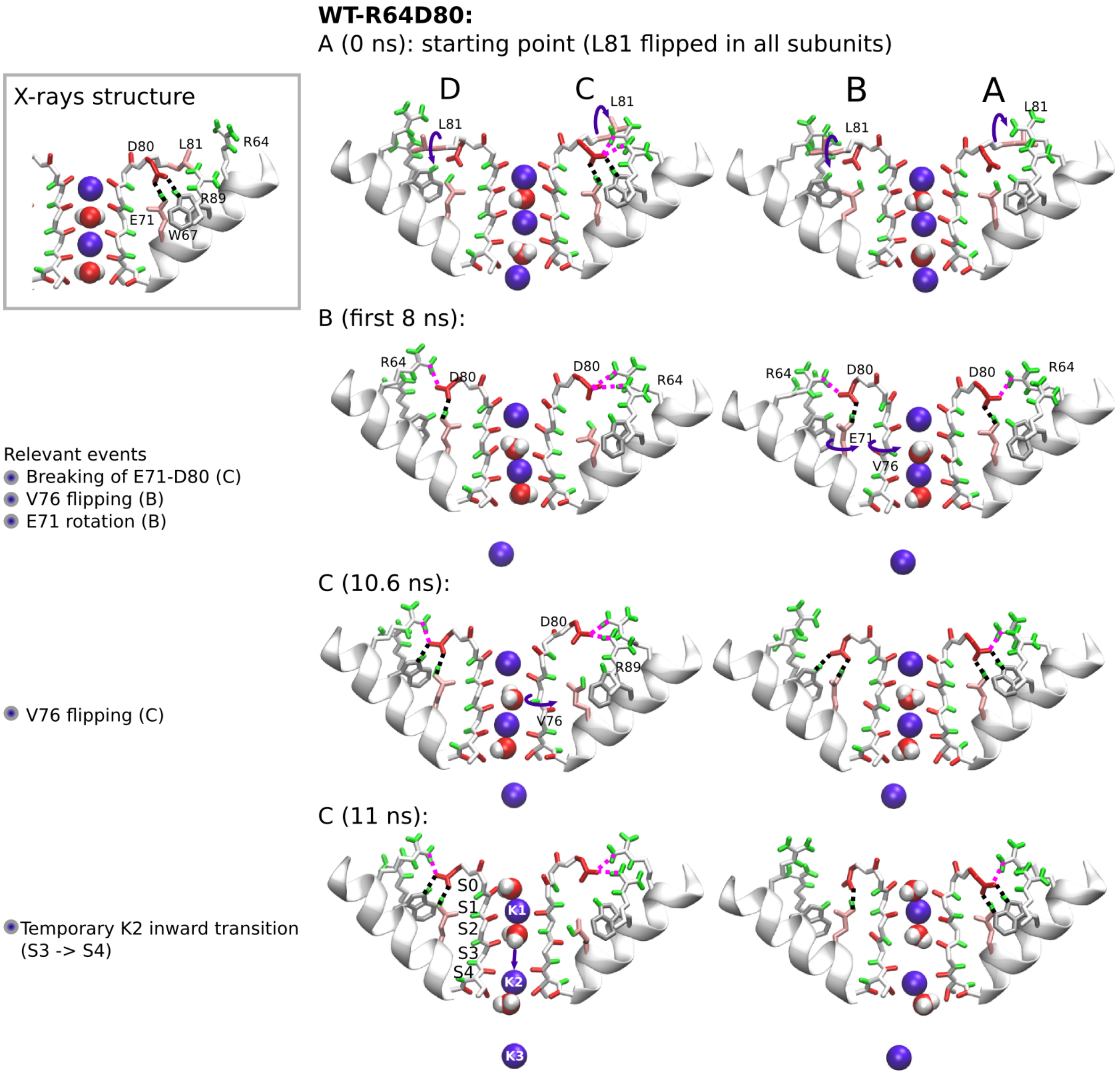

Figure S7. A sequence of events observed in simulation WT-R64D80 is represented by snapshots. The simulation WT-R64D80 ($\approx$ 45 ns) started with R64 residues located near D80 in all the four subunits. The sequence is reported from the starting configuration until the first of a few reverse transitions of the innermost ion K2 (01010+1 $\rightleftarrows$ 01001+1) occurred around 11 ns of the simulation.

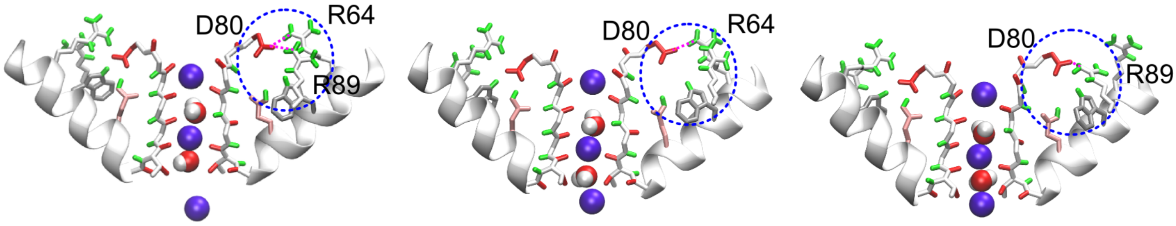

Figure S8. Snapshots from simulation WT-R64D80 show the formation a H-bond between D80 and arginines R64 and R89. The disruption of the E71-D80-W67 linkages and the outward movement of D80 were promoted by the D80–R64 H-bonds and temporarily stabilized by the presence of H-bonds between D80 and R64, or D80 and R89 or D80 and both the arginines.

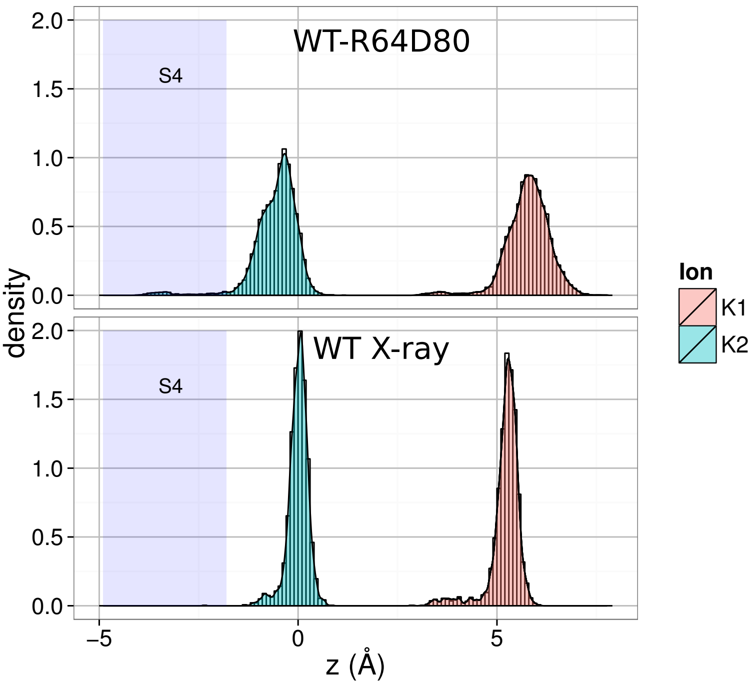

Figure S9. The graphs illustrate the effect of weakening in the spatial definition of the K^+ binding sites in the selectivity filter. The effect in induced by the destabilizing influence of the D80-R64 interaction. Distributions of z-coordinate for two permeating ions (K1 and K2) are shown. The distributions were calculated for identical ion occupancy 01010+1 from i) a simulation of duration 7 ns starting from the X-ray structure with R64 is located relatively far away from D80; and ii) a part of simulation WT-R64D80 (duration 15 ns) with E71-D80-W67 H-bonds were broken as a consequence of D80–R64 interaction.

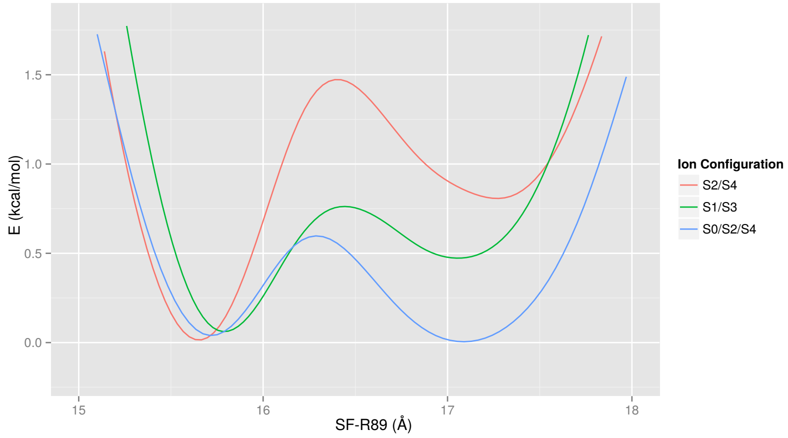

Figure S10. The FES for the dynamics of R89 in the WT is shown as a function of SF − R89 (the distance between the C_ζ atom of R89 and the COM of the selectivity filter) in the cases of different conformations of the K^+ ions in the selectivity filter: S2/S4, S1/S3, and S0/S2/S4. The FES and distance SF − R89 are shown in kcal/mol and in Å respectively.

those delivered by R64 alone. So, R69 plays a facilitating role in the joint action with R64 for breaking E71-D80-W67 linkages.

6.3. Radial distribution functions

Fig. S11 reports the radial distribution functions (RDF) between Cl^- ions and residues R64 and R89. The RDFs confirm the strong interaction between the arginines and negatively charged elements of the extracellular bulk. The functions were estimated on the base of an unbiased simulation of WT.

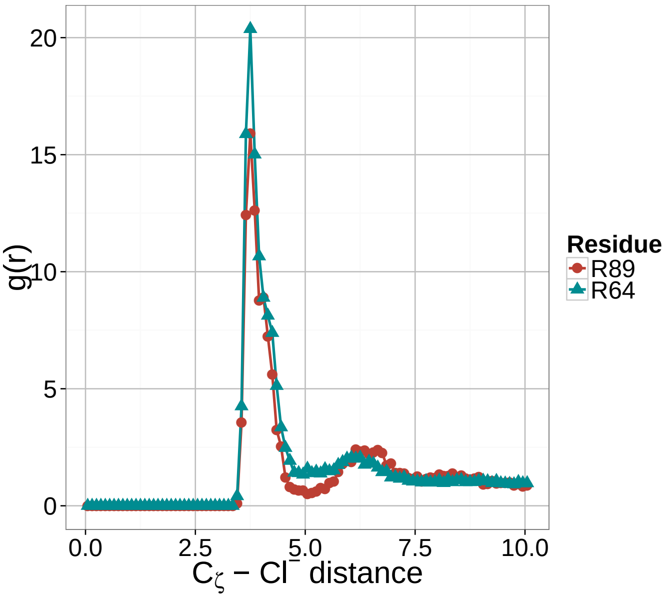

Figure S11. Radial distribution functions (RDF) between Cl^- ions in the bulk and arginines R64 and R89. The RDFs are shown as a function of the distance, $C_\zeta - Cl^-$, between Cl^-ions and the atom C_ζ of each arginines. The distances is shown in Å.

7. Opposite influence of R64 and a K^+ ion bound to S0 on the E71–D80 H-bond

Two different positions of R64 with respect of D80 were obtained by restraining the position of L81 side chain: i) *R64 far* (L81 non-flipped), $\chi1_{81,0} = 297.6°$; ii) *R64 close* (L81 flipped), $\chi1_{81,0} = 185°$. Harmonic restraints were used with $k_{spring} = 5$ kcal/mol degree^2.

The various conformations of the filter content reported in the wt-metaD study, were selected based on extensive preliminary unbiased simulations. The simulations revealed that states with K^+ ions in S2/S4 and no ion in the cavity are more stable than others. In these states K^+ ions from the bulk can enter and leave site S0 on scales of tens of ns. Configurations with K^+ ions bound to S1/S2 had the shortest residence times. If an ion is present in the cavity then the configuration S1/S2 underwent transitions to S0/S2/S3 within 1 ns. In the absence of ion in the cavity, within few ps the configuration S1/S2 first is changed to S1/S3 followed by the transition to S2/S4.

Since configurations with ions in sites S2 and S4 are typically observed in the unbiased simulations, such ions occupancy was chosen in order to avoid additional restraints on ions that might affect the pore dynamics. A set of different K^+ configurations was selected for the free energy calculations according to the K^+ occupancy of S0 (S0/S2/S4 and S2/S4), and further divided depending on the presence of a vacancy in S3: KwKwK and wwKwK (no vacancies); KwK0K and wwK0K (a vacancy in S3). Note that the permeation mechanism which include vacancies have been reported.[30–32]

The total 8 free energy calculations were performed, and the estimated FESs are reported in Fig. S12. The case with a vacancy in site S3 is discussed in the main text. Comparison between calculations performed with or without a vacancy in S3 confirms the similarity of the FESs and,

therefore, the conclusions drawn in the main text. The differences in the FESs should be considered as being dependent on the flipping of V76, which is promoted by the presence of a water molecule in S2 or S3[26]. The latter confirms the existence of a link between D80-region and V76 rearrangements.

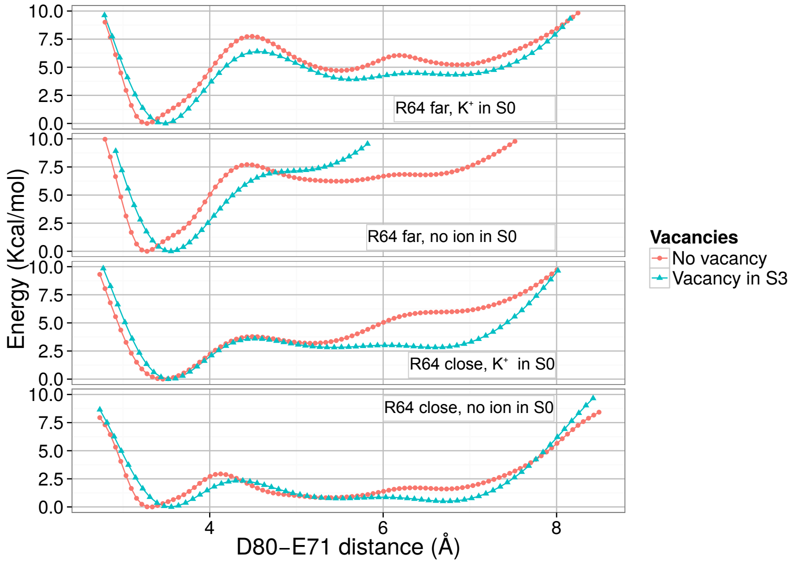

Figure S12. The FESs were obtained by the wt-metaD calculation for to the distance, D80 − E71, between C_γ atom of D80 and the H-bond donor oxygen atom of E71. The FES and distance D80 − E71 are shown in kcal/mol and in Å respectively.

References

1. Phillips, J.C.; Braun, R.; Wang, W.; Gumbart, J.; Tajkhorshid, E.; Villa, E.; Chipot, C.; Skeel, R.D.; Kalé, L.; Schulten, K. Scalable molecular dynamics with NAMD. *Journal of Computational Chemistry* **2005**, *26*, 1781–1802.
2. Essmann, U.; Perera, L.; Berkowitz, M.L.; Darden, T.; Lee, H.; Pedersen, L.G. A smooth particle mesh Ewald method. *The Journal of Chemical Physics* **1995**, *103*, 8577.
3. Fiorin, G.; Klein, M.L.; Hénin, J. Using collective variables to drive molecular dynamics simulations. *Molecular Physics* **2013**, *111*, 3345–3362.
4. Grubmüller, H.; Heller, H.; Windemuth, A.; Schulten, K. Generalized Verlet Algorithm for Efficient Molecular Dynamics Simulations with Long-range Interactions. *Molecular Simulation* **1991**, *6*, 121–142.
5. Tuckerman, M.; Berne, B.J.; Martyna, G.J. Reversible multiple time scale molecular dynamics. *The Journal of Chemical Physics* **1992**, *97*, 1990–2001.
6. MacKerell, A.D.; Wiorkiewicz-Kuczera, J.; Karplus, M. An all-atom empirical energy function for the simulation of nucleic acids. *Journal of the American Chemical Society* **1995**, *117*, 11946–11975.
7. Klauda, J.B.; Venable, R.M.; Freites, J.A.; O'Connor, J.W.; Tobias, D.J.; Mondragon-Ramirez, C.; Vorobyov, I.; MacKerell, A.D.; Pastor, R.W. Update of the CHARMM All-Atom Additive Force Field for Lipids: Validation on Six Lipid Types. *The Journal of Physical Chemistry B* **2010**, *114*, 7830–7843.
8. Noskov, S.Y.; Bernèche, S.; Roux, B. Control of ion selectivity in potassium channels by electrostatic and dynamic properties of carbonyl ligands. *Nature* **2004**, *431*, 830–834.
9. Zhou, Y.; Morais-Cabral, J.H.; Kaufman, A.; MacKinnon, R. Chemistry of ion coordination and hydration revealed by a K^+ channel-Fab complex at 2.0 A resolution. *Nature* **2001**, *414*, 43–48.
10. Grubmüller, H.; Groll, V. Solvate. https://www.mpibpc.mpg.de/grubmueller/solvate (accessed 04/01/2021).
11. Bernèche, S.; Roux, B. The Ionization State and the Conformation of Glu-71 in the KcsA K^+ Channel. *Biophysical Journal* **2002**, *82*, 772–780.

12. Bucher, D.; Guidoni, L.; Rothlisberger, U. The Protonation State of the Glu-71/Asp-80 Residues in the KcsA Potassium Channel: A First-Principles QM/MM Molecular Dynamics Study. *Biophysical Journal* **2007**, *93*, 2315–2324.
13. Zhang, L.; Hermans, J. Hydrophilicity of cavities in proteins. *Proteins: Structure, Function, and Bioinformatics* **1996**, *24*, 433–438.
14. Oostenbrink, C.; Villa, A.; Mark, A.E.; Van Gunsteren, W.F. A biomolecular force field based on the free enthalpy of hydration and solvation: The GROMOS force-field parameter sets 53A5 and 53A6. *Journal of Computational Chemistry* **2004**, *25*, 1656–1676.
15. Cordero-Morales, J.F.; Cuello, L.G.; Zhao, Y.; Jogini, V.; Cortes, D.M.; Roux, B.; Perozo, E. Molecular determinants of gating at the potassium-channel selectivity filter. *Nature Structural & Molecular Biology* **2006**, *13*, 311–318.
16. Cordero-Morales, J.F.; Jogini, V.; Lewis, A.; Vasquez, V.; Cortes, D.M.; Roux, B.; Perozo, E. Molecular driving forces determining potassium channel slow inactivation. *Nature Structural & Molecular Biology* **2007**, *14*, 1062–1069.
17. Cordero-Morales, J.F.; Cuello, L.G.; Perozo, E. Voltage-dependent gating at the KcsA selectivity filter. *Nature Structural & Molecular Biology* **2006**, *13*, 319–322.
18. Chakrapani, S.; Cordero-Morales, J.F.; Perozo, E. A Quantitative Description of KcsA Gating II: Single-Channel Currents. *The Journal of General Physiology* **2007**, *130*, 479 –496.
19. Doyle, D.A.; Cabral, J.M.; Pfuetzner, R.A.; Kuo, A.; Gulbis, J.M.; Cohen, S.L.; Chait, B.T.; MacKinnon, R. The Structure of the Potassium Channel: Molecular Basis of K^+ Conduction and Selectivity. *Science* **1998**, *280*, 69 –77.
20. Fowler, P.W.; Abad, E.; Beckstein, O.; Sansom, M.S.P. Energetics of Multi-Ion Conduction Pathways in Potassium Ion Channels. *Journal of Chemical Theory and Computation* **2013**, *9*, 5176–5189.
21. Buck, M.; Bouguet-Bonnet, S.; Pastor, R.W.; MacKerell, A.D. Importance of the CMAP Correction to the CHARMM22 Protein Force Field: Dynamics of Hen Lysozyme. *Biophysical Journal* **2006**, *90*, L36–L38.
22. Laio, A.; Gervasio, F.L. Metadynamics: a method to simulate rare events and reconstruct the free energy in biophysics, chemistry and material science. *Reports on Progress in Physics* **2008**, *71*, 126601.
23. Laio, A.; Parrinello, M. Escaping free-energy minima. *Proceedings of the National Academy of Sciences* **2002**, *99*, 12562–12566.
24. Barducci, A.; Bussi, G.; Parrinello, M. Well-Tempered Metadynamics: A Smoothly Converging and Tunable Free-Energy Method. *Physical Review Letters* **2008**, *100*, 020603.
25. Bernèche, S.; Roux, B. Molecular Dynamics of the KcsA K^+ Channel in a Bilayer Membrane. *Biophysical Journal* **2000**, *78*, 2900–2917.
26. Bernèche, S.; Roux, B. A Gate in the Selectivity Filter of Potassium Channels. *Structure* **2005**, *13*, 591–600.
27. Domene, C.; Klein, M.L.; Branduardi, D.; Gervasio, F.L.; Parrinello, M. Conformational Changes and Gating at the Selectivity Filter of Potassium Channels. *Journal of the American Chemical Society* **2008**, *130*, 9474–9480.
28. Piccinini, E.; Ceccarelli, M.; Affinito, F.; Brunetti, R.; Jacoboni, C. Biased Molecular Simulations for Free-Energy Mapping: A Comparison on the KcsA Channel as a Test Case. *Journal of Chemical Theory and Computation* **2008**, *4*, 173–183.
29. Chakrapani, S.; Cordero-Morales, J.F.; Jogini, V.; Pan, A.C.; Cortes, D.M.; Roux, B.; Perozo, E. On the structural basis of modal gating behavior in K^+ channels. *Nature Structural & Molecular Biology* **2010**, *18*, 67–74.
30. Furini, S.; Domene, C. Atypical mechanism of conduction in potassium channels. *Proceedings of the National Academy of Sciences* **2009**, *106*, 16074–16077.
31. Jensen, M.O.; Borhani, D.W.; Lindorff-Larsen, K.; Maragakis, P.; Jogini, V.; Eastwood, M.P.; Dror, R.O.; Shaw, D.E. Principles of conduction and hydrophobic gating in K^+ channels. *Proceedings of the National Academy of Sciences* **2010**, *107*, 5833–5838.
32. Jensen, M.O.; Borhani, D.W.; Lindorff-Larsen, K.; Dror, R.O.; Shaw, D.E. Reply to Domene and Furini: Distinguishing knock-on and vacancy diffusion mechanisms. *Proceedings of the National Academy of Sciences* **2010**, *107*, E129–E129.
